# Dissent in the sediment? Lake sediments as archives of short- and long-range impact of anthropogenic activities in northeastern Germany

**DOI:** 10.1007/s11356-023-28210-8

**Published:** 2023-07-03

**Authors:** Marcel Pierre Simon, Marlene Schatz, Leonard Böhm, István Papp, Hans-Peter Grossart, Thorbjørn Joest Andersen, Miklós Bálint, Rolf-Alexander Düring

**Affiliations:** 1 Institute of Soil Science and Soil Conservation, Research Centre for BioSystems, Land Use and Nutrition (iFZ), Justus Liebig University Giessen, Heinrich-Buff-Ring 26-32, 35392 Giessen, Germany; 2 Doctoral School of Chemistry, University of Debrecen, Egyetem Tér 1, Debrecen, 4032 Hungary; 3 Leibniz Institute of Freshwater Ecology and Inland Fisheries, Dept. Plankton and Microbial Ecology, Zur alten Fischerhütte 2, OT Neuglobsow, 16775 Stechlin, Germany; 4 Institute for Biochemistry and Biology, Potsdam University, Maulbeerallee 2, 14469 Potsdam, Germany; 5 Section for Geography, Department of Geosciences and Natural Resource Management, University of Copenhagen, Øster Voldgade 10, 1350 Copenhagen K, Denmark; 6 Senckenberg Biodiversity and Climate Research Centre, Senckenberganlage 25, 60325 Frankfurt am Main, Germany; 7 LOEWE Centre for Translational Biodiversity Genomics, Senckenberganlage 25, 60325 Frankfurt am Main, Germany; 8 Institute of Insect Biotechnology, Justus Liebig University Giessen, Heinrich-Buff-Ring 26-32, 35392 Giessen, Germany

**Keywords:** Organochlorine pesticide (OCP), Dichlorodiphenyltrichloroethane (DDT), Sediment core, Chronology, Trace elements, Mecklenburg-Brandenburg lake district, Landscape development, Transformation products

## Abstract

The suitability of lake sediment cores to reconstruct past inputs, regional pollution, and usage patterns of pesticides has been shown previously. Until now, no such data exist for lakes in eastern Germany. Therefore, 10 sediment cores (length 1 m) of 10 lakes in eastern Germany, the territory of the former German Democratic Republic (GDR), were collected and cut into 5–10-mm layers. In each layer, concentrations of trace elements (TEs) As, Cd, Cr, Cu, Ni, Pb, S, and Zn, as well as of organochlorine pesticides (OCPs), i.e., dichlorodiphenyltrichloroethane (DDT) and hexachlorocyclohexane (HCH), were analyzed. A miniaturized solid–liquid extraction technique in conjunction with headspace solid-phase microextraction (HS-SPME) and gas chromatography–mass spectrometry (GC–MS) was used for the latter. The progression of TE concentrations over time is uniform. It follows a trans-regional pattern and is indicative of activity and policy making in West Germany before 1990 instead of those in the GDR. Of OCPs, only transformation products of DDT were found. Congener ratios indicate a mainly aerial input. In the lakes’ profiles, several regional features and responses to national policies and measures are visible. Dichlorodiphenyldichloroethane (DDD) concentrations reflect the history of DDT use in the GDR. Lake sediments proved to be suitable to archive short- and long-range impacts of anthropogenic activity. Our data can be used to complement and validate other forms of environmental pollution long-term monitoring and to check for the efficiency of pollution countermeasures in the past.

**Supplementary Information:**

The online version contains supplementary material available at 10.1007/s11356-023-28210-8.

## Background

Over the last two centuries, the territory of eastern Germany was subject to a large number of fundamental changes in economic and institutional conditions, e.g., the Industrial Revolution (starting approx. 1815–1835), two World Wars (1914–1918 and 1939–1945), an economic depression (1929–1936), the separation from West Germany (1949), the rise and fall of the German Democratic Republic (GDR; 1949–1990) and the German reunification (1990) along with the integration into the European Union (EU).

These developments affected the population, their activity, and their growth (population size). Humans have always perhaps more than any other species shaped their surroundings to fit their needs, i.e., to assure their livelihood. Apart from urbanization and sealing of soils, the most severe effects on the landscape were due to agricultural activities that were later complemented by industrial activities and led to a general deterioration of the environment (Goudie 2019; Scheffer et al. 2019).

The first large-scale deterioration through agricultural intensification occurred in medieval times, when large areas of wood were systematically deforested (Sommer et al. 2008). After World War 2, intensification was accomplished through industrialization of agriculture. The 1950s were the decade of collectivization of agricultural property in the GDR, so that in 1960, about 85% of farmland was either collectivized or state owned. In the 1970s and 1980s, agriculture developed towards an industrial organized production system. The creation of very large field units (60–100 ha) was accompanied by habitat loss through removal of field boundaries and terraces (Sommer et al. 2008). Additionally, more powerful farm machinery enabled deeper soil tillage practices. All this led to oversized irrigation projects, intensive soil exploitation, and the large-scale use of pesticides and fertilizers among others (Bauerkämper 1993; Radkau 2012). In the late 1970s, a general discourse about ramifications of environmental exploitation started in West Germany and led to countermeasures there (Bauerkämper 2004). In the GDR, however, despite results of several scientific studies, only limited measures for the protection of the environment were taken (Reichelt 1992). It was only after Germany’s reunification that the real extent of environmental damage became clear to the public and only then serious countermeasures were taken (Welford 1991).

In terms of industrial development, the highest priority for the economic lead of the GDR’s regime was to the fulfillment of annual economic plans. Establishing records of production at almost any cost was seen as a guarantee for the regime to stay in power (Buck 2000). The environmental effects of this strategy became obvious at the end of the GDR. It had been leading the ranks of countries polluting the air with SO_2_ and dust emissions for several years, supplemented with high emissions of NO_x_ and hydrocarbons (Buck 1996). Most of these remained inside of the territory and were deposited back to terrestrial and aquatic surfaces in the vicinity of the sources, of which the energy-producing sector was the main emitter. The most affected districts were in the center or south of the GDR, where most of the industry was situated. A significant proportion was exported to neighboring countries downwind (15% in 1988; Buck 1996). Apart from detrimental effects on the health of the population (e.g., respiratory diseases), effects on the environment were enormous. SO_2_ and NO_*x*_ emissions led to acid rain and acidification of soils and vegetation. The ensuing forest dieback (“Waldsterben”) diminished and deforested entire forest districts (Buck and Spindler 1982). Depositions of dust contaminated the soil with heavy metals. Regulatory limits in soils surrounding smelters and accumulator factories were continuously and considerably exceeded (AdW 1990).

The state of the water compartment was similarly disastrous. In particular, the combination of naturally low precipitation rates, very high water consumption, and usage (up to 4 times as much as neighboring countries; Melzer 1985) combined with insufficient to nonexistent wastewater treatment had worsened the situation in aquatic systems. In 1989, almost 50% of all categorized waterways in the GDR were biologically desolated or dead due to pollution. Almost a quarter of all standing waters were unsuited for drinking water generation, and in 54% pollution rendered it unprofitable. One-third of all lakes were incapable of self-cleaning, and this ability was hampered in another third. Only 1% of lakes were biologically intact (DBT 1994).

Understanding the environmental impacts of such agricultural practices and policy making is important. It can benefit the prediction of developments associated with similar practices nowadays. Environmental archives like peat cores, growth rings in trees, ice cores, or lacustrine sediments are used to reconstruct past developments (Waters and Turner 2022). They help understand pre-disturbed conditions and provide long-term monitoring data (Blais et al. 2015), which is especially valuable where other data is scarce or nonexistent (Bálint et al. 2018). An often used proxy indicator for human impacts in paleolimnology studies that utilize these archives is the concentration profile of trace elements (TEs) like As, Cd, Cr, Cu, Ni, Pb, S, and Zn (Krachler et al. 2003; Aliff et al. 2020; Shotyk 2022). They are generally harmful in higher concentrations to plants or animals including human beings (Tóth et al. 2016). Depending on the respective geogenic background, TEs occur naturally in soils and water. Through human (industrial) activity, they can be mobilized, leached and reallocated, and often accumulate to concentrations that pose a threat to plant and animal life (Alloway 2013). Human activities include mining, refining, combustion of fossil fuel, and metallurgy, and they share the atmosphere among others as a common path of emission (Csavina et al. 2011). When emitted into the air, they are mostly adsorbed to particles and can thus be subject to dry and wet deposition following atmospheric transport over short and long distances (Johansson et al. 2001; Shevchenko et al. 2003; Marina-Montes et al. 2020). Telmer et al. (2004) reported that dry deposition contributes significantly within shorter distances of ca. 15 km from an anthropogenic source such as a smelter, whereas wet deposition is the dominant process controlling the deposition of TEs beyond 15 km of the source. Depending on weather, ca. 50% of all emissions are available for long-range atmospheric transport (LRAT) of hundreds to thousands of kilometers (ca. 5.000 km under average weather conditions).

Pesticide records in biological archives can serve as another proxy indicator for human impact. Such records can result from intensification in agricultural activity or—close to production sites—of industrial activity. In the past, organochlorine pesticides (OCPs) have been used intensively, beginning with World War 2. Two very well-known representatives, DDT (dichlorodiphenyltrichloroethane) and lindane (hexachlorocyclohexane (HCH)) were produced and used widely in the 1940s to 1980s (AMAP 1998; van den Berg et al. 2017). Because of their toxicity to non-target organisms and high persistence in the environment, their production and use were restricted and banned following the Stockholm Convention 2001 (UN 2001). Today, they are still found in multiple media all over the world (Li et al. 2014; Camenzuli et al. 2016; Tepanosyan et al. 2020; Olisah et al. 2020). As lipophilic and semi-volatile compounds, they tend to adsorb to organic matter, but can be volatilized and thus be transported and deposited in areas far away from their points of production or application via LRAT. Therefore, they tend to accumulate in colder areas, e.g., Arctic/Antarctic or in high altitudes, where they reach concentrations comparable to source regions (Wania and Mackay 1993; Lee et al. 1998).

The northern part of eastern Germany features a high density of water bodies in a landscape which has been subjected to over 80 years of intensive agriculture (Bauerkämper 2004; Sommer et al. 2008), providing a perfect opportunity for the examination of lacustrine sediment records for TEs and OCPs throughout the history of the GDR and beyond.

While such data was used to reconstruct pollution history in other industrialized countries, e.g., in Russia (Adams et al. 2018), Canada (Kurek et al. 2019), and Switzerland (Chiaia-Hernández et al. 2020), no such data exist for Germany. Therefore, the aim of this work is to find evidence of agricultural and industrial activity of East Germany in dated lacustrine sediment profiles. Ten lakes in northeastern Germany were sampled and dated, and their contents of As, Cd, Cr, Cu, Ni, Pb, S, Zn, DDT and its transformation products (TPs), and HCH determined. The results were intended to shed light on anthropogenic impacts on the environment throughout the last 100 years. Data was evaluated with regard to finding indicators for system changes and possibly cultural and socioeconomic transitions.

## Methods

### Study sites

**Fig. 1:**
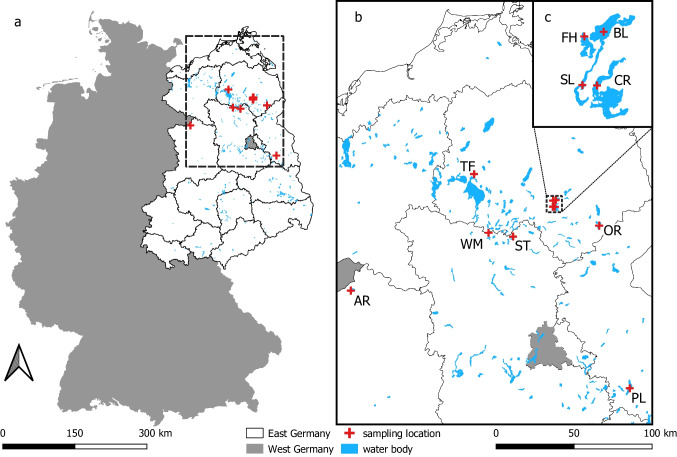
Map of today’s Germany (**a**) divided into West Germany (gray) and East Germany (white) with district borders of the GDR. Blue areas indicate water bodies. Red crosses show sampling locations in the lakes. Section **b** shows a closer view of the sampling locations. Section **c** shows a close-up of the four interconnected lakes FH, BL, SL, and CR. This figure was created with QGIS Desktop 3.16.16 (QGIS Development Team 2022) using open GIS data “vg-hist-001” of the German Federal Agency for Cartography and Geodesy (BKG 2020) and “Waterbodies” of the German Federal Institute of Hydrology (BfG 2021)

Sediments in 10 freshwater lakes in the north of the former GDR’s territory (Fig. 1) were sampled.

All lakes shared common characteristics. They were alkaline with a pH range of 7.5–9, their conductivity varied from 250 to 800 µS·cm^−1^, and they were formed during the last glacial period. Only Lake Arend (AR) is different as the present lake was formed more recently in a sinkhole after suberosion of a salt dome in the years 822 and 1685 (Halbfass 1896). A summary of the lakes’ characteristics is displayed in Table 1.

**Table 1:** List of sampled lakes and their characteristics (UBA 2004), sorted into the former GDR districts they belonged to. Coordinates are given as string expressions and show the sampling location within a lake. Proportions of catchment composition were determined by counting pixels per respective area in catchment maps provided by Geoportal.de the Open Data map service of the German Federal Agency for Cartography and Geodesy (BKG) on 13 May 2019. Meadows and fields were combined to agricultural area, as it was not possible to discern a difference between them with sufficient certainty in the map data. Waterbodies were excluded from the calculations

Lake	Abbrev	Latitude	Longitude	Surface area (km^2^)	Maximum depth (m)	Catchment area (km^2^)	Catchment composition		
							Forest	Agriculture	Settlement
Neubrandenburg district									
Feldberger Haussee	FH	+ 53.351516	+ 013.439390	1.31	12.5	5.3	59%	11%	31%
Breiter Luzin	BL	+ 53.354081	+ 013.463740	3.45	58.3	12	40%	58%	2%
Schmaler Luzin	SL	+ 53.315475	+ 013.433281	1.45	33.5	29.5	32%	61%	7%
Carwitzersee	CR	+ 53.314518	+ 013.451346	7.22	42.2	53.6	36%	59%	4%
Tiefwarensee	TF	+ 53.529629	+ 012.690718	1.41	23.6	21.9	28%	41%	31%
Oberückersee	OR	+ 53.189547	+ 013.864672	5.89	24.5	23	37%	53%	10%
Frankfurt (Oder) district									
Scharmützelsee	PL	+ 52.242076	+ 014.048144	12.1	29	112	56%	35%	10%
Magdeburg district									
Arendsee	AR	+ 52.888519	+ 011.460285	5.14	48.7	29.8	37%	56%	8%
Potsdam district									
Stechlin	ST	+ 53.157715	+ 013.034102	4.25	68	12.4	99%	0%	1%
Wummsee	WM	+ 53.187020	+ 012.800844	1.48	36	14	92%	7%	1%

### Sediment core collection and sample preparation

Sediment cores were collected with a gravity corer (90-mm diameter; UWITEC, Mondsee, Austria) at the deepest points of the lakes, where sediment accumulation is strongest (Blais and Kalff 1995; Table 1). The core length of 1 m was deemed enough to cover a time span of at least 100 years starting from the year of sampling, i.e., the year 1915, which was on average located between 210-mm and 510-mm sediment depth, depending on the lake. Coring was performed in August 2015 (all but one lake) and December 2016 (Lake Stechlin, ST). Cores were transported to the lakeshore, and were immediately subsampled by slicing with a designated core slicer (UWITEC). Sediment slices were 5 mm (Lakes Feldberger Haussee, FH, and Breiter Luzin, BL), or 10 mm in thickness (all other lakes) to account for different sedimentation rates, which depend on lake trophy and biochemical processes (Ahn 2018). Samples were transported to the laboratory at 4 °C, weighed, and immediately frozen at − 20 °C until further processing.

### Loss on ignition

Sediment weight loss on ignition (LOI) was analyzed according to the method of Nelson and Sommers (1996) described in Bensharada et al. (2022). Crucibles were weighed without sample (WC), and samples were dried in crucibles overnight at 105 °C. After cooling, crucibles with dry samples were re-weighed (WS). For determination of the organic matter content, samples were combusted in an electric muffle furnace (SNOL 8,2/1100, Utena, Lithuania) at 500 °C for 4 h. After cooling in a desiccator, the samples were reweighed (WA). LOI was calculated as $\frac{\mathrm{WS}-\mathrm{WA}}{\mathrm{WS}-\mathrm{WC}}\times 100$.

### Radioisotope dating and cross-correlation

Cores from Lakes BL, Schmaler Luzin (SL), Carwitzer (CR), Tiefwaren (TF), Oberucker (OR), Scharmützel (PL), ST, and Wumm (WM) were dated with ^210^Pb and ^137^Cs radioisotopes. Sediment horizons were freeze-dried before radioisotope dating. Dating was performed by direct gamma assay of the isotopes of 1 g of freeze-dried samples at the Department of Geosciences and Natural Resource Management of the University of Copenhagen. A constant rate of supply (CRS) dating model was applied to construct ^210^Pb chronologies (Appleby and Oldfield 1978). These models were independently verified with ^137^Cs.

Between the cores from lakes BL and FH, the concentrations of several elements were highly correlated (Online Resource: Fig. S1). These correlations were used to align the two cores, and the age model of BL was applied to establish the FH chronology.

For lake AR, the LOI values were aligned with the organic matter (OM) content of another core for which an age model was already published (Rothe et al. 2015). Both of these cores were taken close to the deepest point of lake AR. The chronology of the AR core was established based on this alignment (Online Resource: Fig. S2).

In lakes BL and TF, the initial age model suggested the presence of peaks of DDT derivatives in the nineteenth century. This is inconsistent with loads observed in all other lakes, and with the onset of DDT application in the twentieth century. In addition, lake TF received a hypolimnic treatment with aluminum between 2001 and 2005 as a restoration measure from eutrophic to mesotrophic states (Wauer et al. 2009; Rösel et al. 2012). The original age model would place the Al peak of the treatment in the 1970s. Fallout peaks of ^137^Cs in 1986 (Chernobyl disaster) and 1962/1963 (peak of nuclear bomb tests in the high atmosphere) were re-evaluated, and adjusted the age model after considering these inconsistencies (Online Resource: Fig. S3).

### Microwave-assisted aqua-regia extraction

Acids used for microwave-assisted aqua-regia extraction (MAE-AR) were of analytical-reagent grade. Nitric acid (HNO_3_, 69% (w/v)) and hydrochloric acid (HCl, 35% (w/v)) were purchased from Merck KGaA (Darmstadt, Germany) and Carl Roth GmbH & Co. KG (Karlsruhe, Germany), respectively. Ultra-pure water (mQ) was obtained through filtering of deionized water with Milli-Q A10 water purification system (Merck KGaA) and was used for all experiments.

A StarT-1500 microwave (MLS GmbH, Leutkirch, Germany) was used to perform MAE-AR, which holds up to 10 polytetrafluorethylene (PTFE) digestion vessels with a volume of 100 mL each. A modified US EPA method (3051A (SW-846); US EPA 2007) as described by Öztan and Düring (2012) was applied. In brief, 0.3 g of freeze-dried sediment sample was weighed directly into the PTFE vessels, 6 mL HCl (35%) and 2 mL HNO_3_ (69%) were added, and the microwave program as described in the Online Resource (Table S1) was run. Following extraction and cooling, extracts were transferred to 50-mL calibrated polypropylene flasks, pretreated with HNO_3_. Extracts were made up to volume with deionized water, filtered (185 mm; Macherey–Nagel MN 280 1/4), and stored in polyethylene bottles at 4 °C until analysis. Blanks were subjected to the same extraction procedure as samples were. To avoid cross contamination of PTFE vessels with TEs from previous extractions, vessels were cleansed in between each sample extraction using 10 mL HNO_3_ (69%) and the same MAE-AR program as used for samples.

### Inductively coupled plasma–optical emission spectrometry analysis

Concentrations of elements in sediments were measured using an inductively coupled plasma–optical emission spectrometer (ICP–OES; Agilent 720ES, Darmstadt, Germany) with axial torch and echelle optic configuration, charge couple device (CCD) detection system, and full wavelength coverage from 167 to 785 nm. Operating parameters were as follows: Incident power was 1.20 kW, and plasma gas and auxiliary gas flow were 16.5 and 1.5 L·min^−1^, respectively. Sample uptake and test time per repetition were 45 and 30 s, respectively. Element calibration solutions were produced by dilution of ICP standards (Carl Roth GmbH & Co. KG). Although more elements were measured, the considerations in this work were limited to the elements As, Cd, Cr, Cu, Ni, Pb, S, and Zn, as these were deemed most suitable for evaluating industrial impact (Alloway 2013; Mills et al. 2017).

### Miniaturized solid–liquid extraction of OCPs

Organic solvents acetone and methanol (both gradient grade for HPLC) were purchased from VWR International (Radnor, PA, USA), and petroleum ether (40–60 °C, p.a.) was purchased from Merck KGaA. Analytical standards (purity) were used for calibration or as internal standards if isotopically labeled: 2,4’-dichlorodiphenyldichloroethane (DDD, 97.5%), 2,4’-dichlorodiphenyldichloroethylene (DDE, 99%), 2,4’-DDT (99.5%), 4,4’-DDD (99.5%), 4,4’-DDE (98%), 4,4’-DDT (99.5%), ^13^C-2,4’-DDT (100%), and γ-HCH (98.6%) were purchased from Dr. Ehrenstorfer GmbH (Augsburg, Germany). α-HCH (≥ 98%) and δ-HCH (≥ 98%) were purchased from Sigma-Aldrich (St. Louis, MO, USA). β-HCH (99.5%) was obtained from the Institute of Industrial Organic Chemistry (Warsaw, Poland). 4,4’-DDD-D_8_ (99.7%), 4,4’-DDE-D_8_ (99.4%), and α-HCH-D_6_ (99.2%) were purchased from CDN Isotopes (Pointe Claire, Canada). ^13^C-4,4’-DDT (99%) was purchased from the Cambridge Isotope Laboratories Inc. (Andover, MA, USA). Purity was considered when preparing stock solutions of standards.

Samples were extracted based on a miniaturized solid–liquid extraction method (MISOLEX; Simon et al. 2021). In brief, 0.5 g of freeze-dried sediment sample was weighed in a 20-mL clear-glass head-space vial. Five milliliters of acetone and 5 mL of petroleum ether were added, and the vial was closed tightly with a screw cap. The sample was shaken in a horizontal shaker for 30 min at 200 rpm (Swip KS-10, Edmund Bühler GmbH, Bodelshausen, Germany) and then centrifuged for 10 min at 1000 rpm (207.2 g; Rotanta 460 R, Hettich AG, Bäch, Switzerland). The supernatant was transferred into a 20-mL brown-glass head-space vial. Another 10 mL of petroleum ether was added to the sample, and the process was repeated. The supernatant was added to the one taken before, resulting in approx. 12 mL of extract. An aliquot of 10 mL was transferred to a fresh 20-mL brown-glass head-space vial, and 2 µL of internal standard mix (Online Resource: Table S2) was added, equivalent to a concentration between 1 and 3 ng·mL^−1^ in the final sample. The extract was evaporated to dryness under a gentle stream of nitrogen. Immediately after evaporation, 100 µL of methanol, serving as solubilizer, and 10 mL of salt solution (200 g NaCl in 1 L ultrapure water) were added.

### SPME and GC–MS analysis

Analysis of OCPs in sediment samples was carried out with a Trace GC Ultra gas chromatograph (Thermo Fisher Scientific, San Jose, CA, USA), a CombiPAL autosampler (CTC Analytics AG, Zwingen, Switzerland) equipped with a SPME fiber assembly, and an ITQ 900 mass spectrometer (Thermo Fisher Scientific). For all measurements, a SPME fiber coated with PDMS (100 µm) was used (Sigma-Aldrich, St. Louis, MO, USA). Extraction by SMPE of prepared samples started with a heat up phase in the agitator for 5 min to 80 °C, followed by headspace extraction at the same temperature for 30 min. After extraction, the fiber was thermally desorbed in splitless mode in the GC injector for 3 min, after which it switched back to a split flow of 30 mL·min^−1^. At the start and end of each SPME sample cycle, the fiber was desorbed in a needle heater for 5 min at 270 °C. Chromatographic separation was conducted on a fused silica capillary column (TG-XLBMS 60 m, 0.25-mm inner diameter, 0.25-μm coating thickness; Thermo Fisher Scientific). Helium (≥ 99.999%, Praxair, Danbury, CT, USA) was used as carrier gas at a constant flow of 1.0 mL·min^−1^. The initial oven temperature was set at 90 °C and held for 3 min. The temperature was ramped to 150 °C at a rate of 15 °C·min^−1^. Then, it was ramped to 280 °C at a rate of 5 °C·min^−1^ and held for 3 min. Quantification was done in selected ion monitoring (SIM) mode based on one target and one qualifier ion. A list of ions and retention times used is available in the Online Resource (Table S3). The peak areas of analytes in sediment samples were corrected with their respective internal standard (see caption of Table S3). The respective concentration was determined by interpolation of the relative peak areas for each pesticide to standard peak areas of the calibration curve.

### Congener ratios

In this study, DDD was used as indicator for direct input of DDT formulation into the lake, followed by transformation to DDD in the mostly anaerobic sediment. In contrast, DDE was deemed indicative of erosive transport into the sediment following transformation in aerobic conditions in the topsoils outside the lake body. DDD/DDE ratios were calculated of 4,4’ congeners only, as these were found in higher concentrations and thus more consistently throughout the length of the profiles. For the same reason, 4,4’/2,4’ ratios were calculated only of DDD. The technical mixture of DDT contains about 63–80% 4,4’-DDT and 15–21% 2,4’-DDT, resulting in ratios between 3 and 5.1 (Braun et al. 1999; Ricking and Schwarzbauer 2012), while in higher quality mixtures, the 4,4’-DDT proportion is higher.

### Data analysis

To generate mean values that span over several lakes and years, the following method was applied: For each analyte (TE or OCP) and lake separately, values were normalized to the maximum value, which was set to 1. Then, normalized values of a single element of all lakes were put together, ordered according to their date, and then, means covering all values in 5-year spans were calculated. Lakes with a higher sedimentation rate had thicker sediment layers per year than those with lower rates. This means that for the given core length of 1 m, layers from lakes with higher sedimentation rate cover a smaller time period and also yield more data points per 5-year period than the latter. Consequently, for the 5-year periods, not always the same number of points or lakes was covered and sometimes lakes were covered twice. Tables S4 and S5 in the Online Resource provide an overview of data points per 5-year period and which lakes were included.

Land use area proportions in catchment areas of the lakes were analyzed graphically by pixel area counting of map excerpts using Paint.NET Version 4.1.6 (dotPDN LLC). Maps were taken from Geoportal.de, the Open Data map service of the Federal Agency for Cartography and Geodesy (BKG) on 13 May 2019. Meadows and fields were combined to agricultural area, as it was not possible to discern a difference between them with sufficient certainty in the map data. Waterbodies were excluded from the calculations.

### Data storage

Data for this study were published open access (Simon et al. 2023). Additional figures and tables mentioned in the text are available as part of the Online Resource on the article’s webpage (Supplementary file 1; PDF).

## Results

### Radioisotope dating

The dating reasonably covered the time period of interest (1915–2015). Well-resolved ^137^Cs peaks were found in the profiles, indicating the nuclear weapon testing in 1963, which was used to correct the ^210^Pb dating. Figures of age profiles for each lake showing activities of the isotopes per depth are available in the Online Resource (Fig. S4).

### Trace element concentrations

#### Lakes in general

**Fig. 2:**
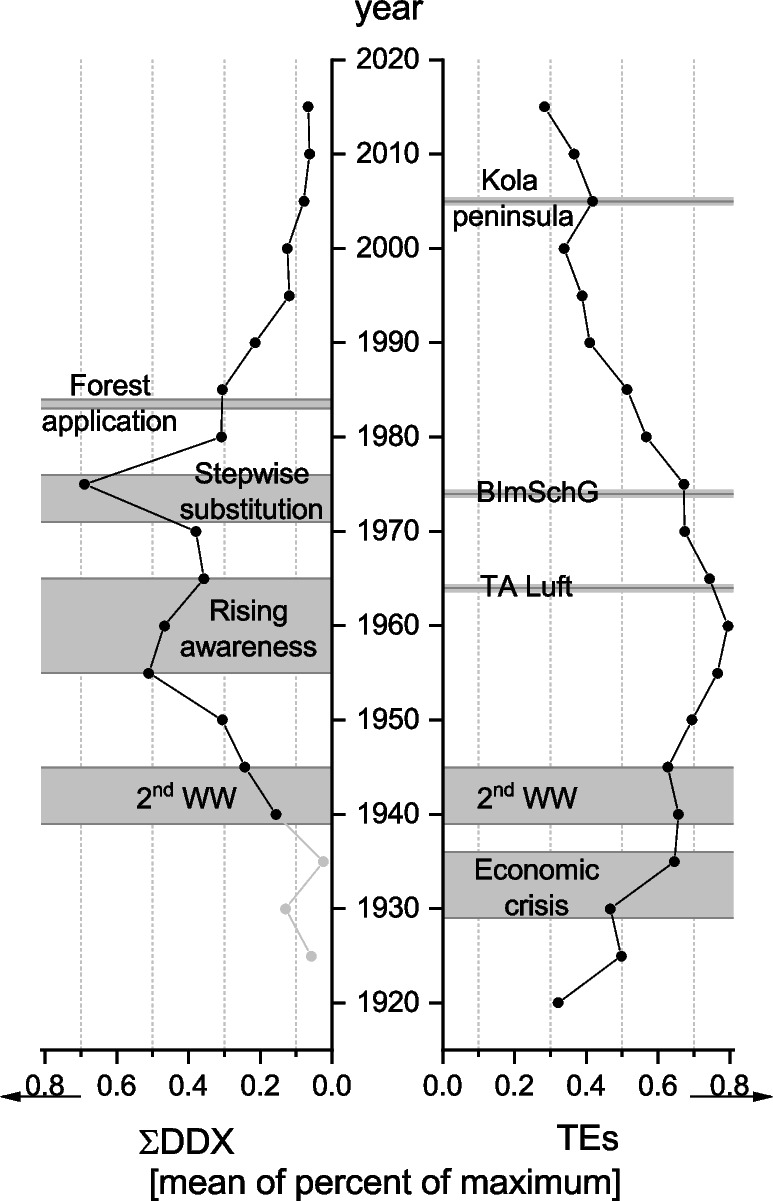
Five-year means of concentrations of ΣDDX (left) as well as TEs (right) of all 10 lakes normalized to the respective maximum of each sum parameter in each lake (excluding lake WM for ΣDDX). A detailed explanation about the calculation is given in the text (Sect. Data analysis). Light gray points depict implausible measurement results that are probably caused by carry-over during sampling. TA Luft (1964) and BImSchG (1974) were the first and second implemented air emission control regulations in West Germany, respectively. This figure was created with OriginPro 2022b (OriginLab Corp., Northampton, MA, USA)

**Fig. 3:**
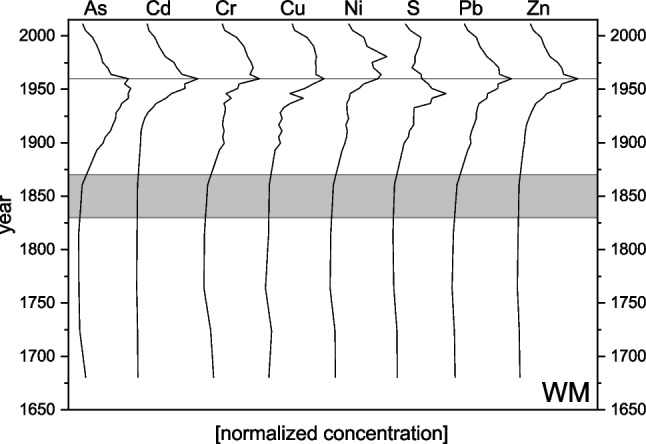
Plot showing normalized concentrations of TEs As, Cd, Cr, Cu, Ni, S, Pb, and Zn in lake WM relative to the maximum concentration of each TE, which was set to 1. The gray area depicts the onset period of the Industrial Revolution in Germany. The gray line denotes the year 1964, when the first air pollution control regulation (TA Luft 1964) was implemented in West Germany. This figure was created with OriginPro 2022b (OriginLab Corp.)

Normalized mean values covering all lakes and elements (As, Cd, Cr, Cu, Ni, Pb, S, Zn) are shown in Fig. 2. Elemental profiles of each lake are available in the Online Resource (Figs. S5–S14). A prominent general pattern is visible: Values are increasing from 1920 from higher than background concentrations until the 1960s, which demarks a turning point. Thereafter, values are decreasing until the year 2000, with a slightly stronger slope from 1975. Concentrations start to rise again until they reach a second maximum in 2005, after which values from before are reached in 2010 and continue to decline until the most recent layers of the cores (ca. 2015). Lakes SL, CR, PL, ST, and WM include data that precede 1900 (Fig. 3 and Online Resource: Figs. S15–S17). In their profiles, elemental concentrations show strong increases starting from assumed background concentrations at around 1850 and 1870 which reach a first peak or plateau at ca. 1900 and develop as described before. Profiles of elements Al, Cr, Ni, and Co show a remarkable resemblance throughout the sampled lakes.

#### Traits of specific lakes

Table 2 shows minimum and maximum concentrations, and the year of the maximum for each lake and element between 1925 and 2015. Lakes ST and WM show the highest ranges of elemental concentrations, followed by lakes SL, CR, AR, and TF. Generally, the elemental concentrations of lakes FH, BL, PL, and OR are the lowest of all 10 lakes. Profiles from lake FH and its neighboring lake BL (Online Resource: Figs. S1, S5, and, S6) show a remarkable resemblance, not only in behavior but also in concentration. This holds true for the pairs SL–CR and ST–WM, albeit with less similarity. Regarding single elements, noticeably high concentration peaks are present for As in lake TF in ca. 1965 (87 mg·kg^−1^) and for Cu in lake ST in ca. 1981 (313 mg·kg^−1^). Considering all profiles, the median of the maximum concentrations of TEs follows this order: S (27,150 mg·kg^−1^) > Zn (379 mg·kg^−1^) > Pb (170 mg·kg^−1^) > Cu (41 mg·kg^−1^) > As (33 mg·kg^−1^) > Cr (15 mg·kg^−1^) > Ni (15 mg·kg^−1^) > Cd (3 mg·kg^−1^).

**Table 2:** Minimum and maximum concentration in mg·kg^−1^ and year of maximum of each element and lake for the period from 1925 to 2015

	As			Cd			Cu			Cr			Ni			Pb			S			Zn		
Lake	Min	Max	Year	Min	Max	Year	Min	Max	Year	Min	Max	Year	Min	Max	Year	Min	Max	Year	Min	Max	Year	Min	Max	Year
Neubrandenburg district																								
FH	0.15	14.5	1953	0.08	1.98	1953	5.74	39	1986	0.74	8.47	1953	1.23	7.16	1986	2.07	108	1953	1,274	15,603	1953	8.92	171	1953
BL	3.94	14.6	1953	0.21	1.96	1953	7.21	16.8	1976	2.59	7.97	1953	3.08	7.01	1953	12.5	102	1959	4,465	16,132	1953	33.6	169	1953
SL	8.95	32.8	1960	0.37	4.7	1960	25	39.8	1960	8.51	17.5	1960	6.26	14	1960	50	232	1960	10,000	566,689	1969	150	397	1960
CR	10.1	33.4	1942	1.25	5.39	1957	27.5	42	1957	14.3	37.4	1957	13.4	26.1	1957	81	279	1968	15,000	34,587	1968	180	403	1968
TF	6	88	1965	0.48	2.3	1971	25	53	1984	8	15.5	1984	7	15	1971	35	140	1971	10,500	21,500	1984	90	360	1984
OR	1.5	6	1965	0.1	0.8	1953	6	18	1982	3.5	10	1977	4	12.5	1959	6	37.5	1953	5,500	11,500	1965	25	85	1953
Frankfurt (Oder) district																								
PL	2.5	8.75	1958	0.2	1.4	1951	16.6	23	1964	3.5	8	1958	4.5	7	1983	22	92	1958	11,700	23,500	1970	60	150	1958
Magdeburg district																								
AR	*n.d.*	33	1954	*n.d.*	4	1974	9	50	1974	2	15	1974	5	22	1974	1	200	1974	6,300	30,800	1958	*n.d.*	500	1974
Potsdam district																								
ST	12	68	1966	1	9	1947	30	300	1981	4	25	1966	6	20	1966	50	550	1947	9,800	33,000	1927	140	1,400	1966
WM	15	66	1951	2.5	12.43	1960	25	54	1960	10	24.6	1960	7.5	21.7	1980	150	649	1960	16,500	47,270	1946	230	960	1960
Median	4.97	32.9	1956	0.29	3.15	1955	20.8	40.9	1975	3.75	15.3	1960	5.5	14.5	1969	28.5	170	1960	9,900	27,150	1962	75	379	1960

*n.d.* not detected

### Organochlorine pesticide concentrations

#### Lakes in general

As only the lower half of the lake WM core was analyzed for OCPs and thus covers a time frame from ca. 1680 to only ca. 1955, it was left out of all further calculations. Of the analyzed OCPs, only 2,4’-DDD, 4,4’-DDD, 2,4’-DDE, and 4,4’-DDE were found. Consequently, ΣDDX denotes the sum of these four congeners. Figure 2 shows 5-year normalized mean values of ΣDDX, calculated for all lakes and congeners. Disregarding implausible values from earlier dates, concentrations started to rise in 1935 and reached a first peak in the mid-1950s (ca. 50% of maximum). After a short decline until 1965, concentrations sharply rose until the main peak in 1975 (ca. 70% of maximum), before they dropped to concentrations before the second peak and, thereafter, continued to decline. Concentrations levelled off at ca. 10% of maximum in 2010.

#### Traits of specific lakes

Table 3 shows minimum and maximum concentrations, and the year of the maximum for each lake and congener between 1925 and 2015. Maximum concentrations of congeners follow this order: 4,4’-DDD > 4,4’-DDE > 2,4’-DDD ≫ 2,4’-DDE. 2,4’-DDE concentrations were by far the lowest of the four congeners, constantly staying below 10 µg·kg^−1^, and in the case of lakes FH and OR below the limit of detection (LOD).

**Table 3:** Minimum and maximum concentration in µg·kg^−1^ and year of maximum of each DDT transformation product for the period from 1925 to 2015. Lake WM is excluded as its data only covers the time frame until ca. 1955

	4,4’-DDD			2,4’-DDD			4,4’-DDE			2,4’-DDE			ΣDDD			ΣDDE			ΣDDX		
Lake	Min	Max	Year	Min	Max	Year	Min	Max	Year	Min	Max	Year	Min	Max	Year	Min	Max	Year	Min	Max	Year
Neubrandenburg district																					
FH	8	38	1963	3	19	1963	5	28	1963	*n.d.*	*n.d.*	*n.d.*	9.86	57.4	1963	4.79	27.8	1963	11.5	170	1963
BL	1.5	36.5	1953	0.1	5	1958	1.1	20	1964	1.3	2.4	1958	1.58	40.5	1953	2.40	22.2	1964	4.80	119	1959
SL	8.5	105	1960	13	67.5	1969	3	70.5	1969	3.3	6.8	1977	8.53	173	1969	10.9	78.3	1969	20.4	251	1969
CR	18	130	1977	11	45	1977	1.3	53	1968	1.0	2	1977	–^*^	175	1977	1.25	55	1968	1.25	228	1977
TF	8.5	204	1984	5	78	1984	6	59	1984	3.0	6.5	1984	13.6	282	1984	6	65.1	1984	24.2	346	1984
OR	1.5	19	1959	*n.d.*	*n.d.*	*n.d.*	1.3	13	1965	0.6	1.9	2013	1.46	18.8	1959	1.96	12.9	1965	1.96	30.2	1965
Frankfurt (Oder) district																					
PL	5.5	86	1977	2.5	30	1977	0.5	48	1977	0.25	1.6	1977	6.63	116	1977	4.64	49.8	1977	10.7	166	1977
Magdeburg district																					
AR	4	235	1974	17	58	1974	2.5	83	1974	1.6	4.4	1974	–^*^	350	1974	3.77	87.3	1974	3.77	437	1974
Potsdam district																					
ST	4.5	140	1966	3.4	55	1966	1.2	60	1966	0.1	4.3	1966	4.47	194	1966	5.42	64.6	1966	9.89	259	1966
WM	–	–	–	–	–	–	–	–	–	–	–	–	–	–	–	–	–	–	–	–	–
Median	5.5	105	1966	5	50	1972	1.3	53	1969	1.15	3.35	1977	6.63	173	1969	4.64	55	1968	9.89	228	1969

*n.d.* not detected

^*^Because of limited data points, a plausible minimum is not present

Among the 10 studied lakes, highest concentrations of ΣDDX were found in lake AR (ca. 380 µg·kg^−1^, ca. 1974) and lake TF (ca. 350 µg·kg^−1^, ca. 1985). Lower concentrations (225–250 µg·kg^−1^) were present in lakes ST, SL, and CR (ca. 1966, 1969 and 1977, resp.). Whereas the lowest concentrations (30–165 µg·kg^−1^) were found in lakes FH, BL, OR, and PL (ca. 1963, 1959, 1965 and 1977, resp.).

**Fig. 4:**
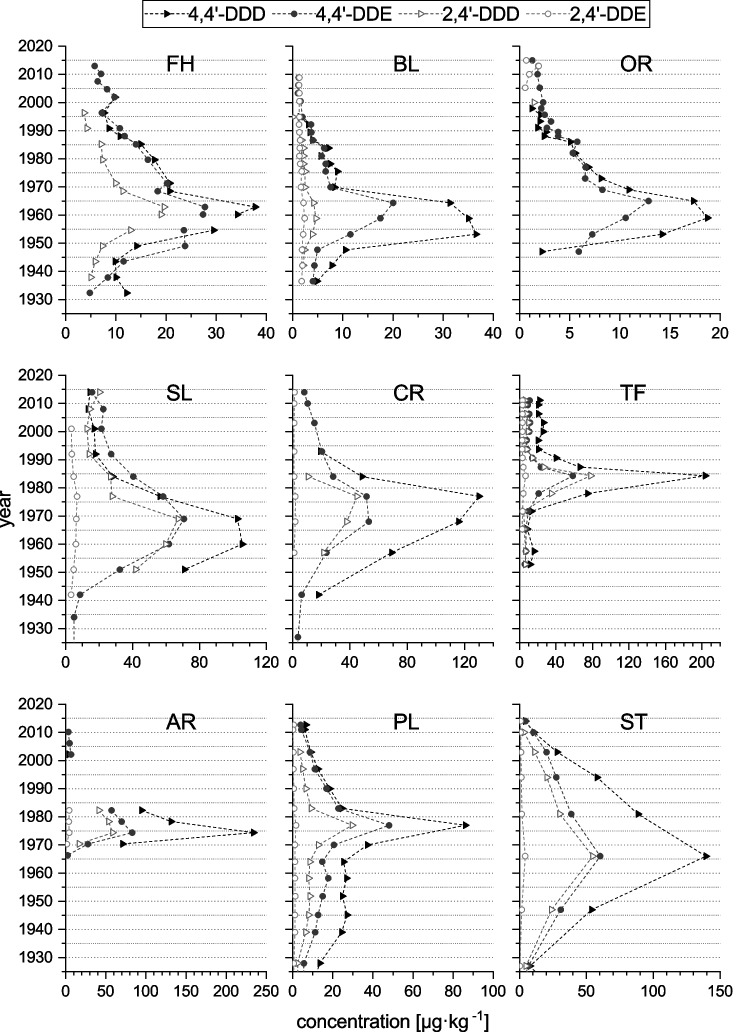
Concentrations of transformation products of DDT in µg·kg^−1^ in each lake profile (excluding lake WM) between the years 1930 and 2015. Note the different *x*-axes. This figure was created with OriginPro 2022b (OriginLab Corp.)

In the single lake profiles (Fig. 4), three points in time of increased concentrations are visible, mirroring the averaged profile of the lakes of Fig. 2: The first is between 1955 and 1965, especially prominent in lakes FH, BL, OR, and PL, while in lake SL, it is 5 years later. The second event is in the 1970s and can be seen in lakes AR, CR, and PL. Lake TF is the only lake where the third event in ca. 1984 is clearly visible, as it constitutes the main peak. Much less distinct indications of this event are present in lakes BL and OR. Lakes ST and WM show a similar behavior compared to TEs (data of WM not shown), where concentrations slowly rose until maximum in the late 1960s followed by a likewise slow decrease to very low concentrations at the top in ca. 2015. However, the very coarse time resolution of these cores could have masked any short-term developments of OCP concentrations.

#### Congener ratios

**Fig. 5:**
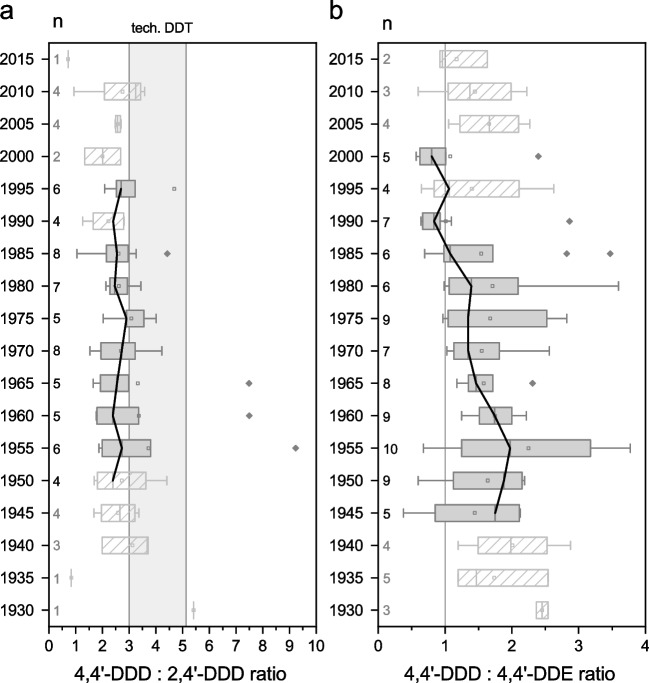
Box plots of 4,4’-DDD/2,4’-DDD (**a**) and 4,4’-DDD/4,4’-DDE (**b**) congener ratios of 5-year periods. *n* is the number of different lakes that are included in a box plot. The gray area in **a** depicts the ratio range which is typical for technical DDT mixtures. High-quality formulations feature higher ratios. The gray line in **b** marks a ratio of 1. Values above 1 are indicative of a higher influence of direct DDT input, while those below 1 indicate a higher proportion of erosive input in the form of DDE. Light gray bordered and hatched unfilled box plots have to be considered with caution, either because only a limited number of lakes is represented, or because the occurrence of DDX at that time is not considered historically sound. This figure was created with OriginPro 2022b (OriginLab Corp.)

Since only TPs of DDT were found, ratios were calculated between them to assess the quality of the used product (4,4’-DDD/2,4’-DDD), as well as to distinguish between direct and erosive input (4,4’-DDD/4,4’-DDE).Box plots summarizing the ratios of all studied lakes in the 5-year periods are depicted in Fig. 5. Light gray, hatched box plots show values that have to be considered with caution, either because only a limited number of lakes is represented, or the occurrence of values at that time is not considered historically sound (see “Data analysis” section). 

4,4’-DDD/2,4’-DDD ratios stay mostly between values of 2 and 3 over the length of the observed period from ca. 1950 to ca. 1995 (Fig. 5a). Ratios of 4,4’-DDD/4,4’-DDE are mostly above 1 until ca. 1985. The median of ratios peaks in ca. 1955 after which it declines until ca. 1970. Then, it slowly increases, reaching a secondary peak in ca. 1980, and drops beneath 1 in ca. 1990. In ca. 1995, it shortly peaks slightly above 1, and returns to almost the same value in ca. 2000.

## Discussion

### Trace elements

The fact that the TE concentrations in sediment cores of different lakes show a very similar behavior among each other leads to the conclusion that they were mainly introduced via diffuse atmospheric deposition. Pacyna (1984) estimated the atmospheric emission of TEs from anthropogenic sources in Europe per country for the year 1979. The order of emitted element concentrations from highest to lowest was Zn > Pb > Cu > As > Cr > Ni > Cd. This is similar to the order found throughout all lakes in this study, indicating that besides the geogenic background, the atmosphere or rather anthropogenic industrial activity is the primary source. Still, an influence of other anthropogenic activities in the vicinity of the lakes cannot be completely excluded. This includes direct run-off from settlements or input from treated or untreated wastewater and other surrounding waterbodies for example, although no clear indications were discernible for most lakes. The only exception is lake ST, which has been impacted by the nuclear power plant Rheinsberg (see below).

Compared to the average geogenic background of the respective federal state in which a lake is situated (LABO 2003), the concentrations of the elements found in the lake sediments were mostly higher (factors ca. 2–15), when taking the period of industrial activity into account. Where the concentrations are lower, at least a trend is visible. In lakes ST and WM, concentrations of Cd, Cr, Pb, and Zn rise above background levels starting from ca. 1893. For both lakes, there are reports of local glass-producing industry in the nineteenth century dumping substantial amounts of ash from wood burning into the lakes, which could be an explanation (Casper et al. 1985). Additionally, the visible increase starting in the second half of the nineteenth century is present for most of the elements in the extended profiles and is probably due to the onset of industrialization in general that has been reported in other studies from European lakes (e.g., Gunten et al. 1997; Shotyk et al. 2002; Thevenon et al. 2011; Elbaz-Poulichet et al. 2020), and which has been proposed as the beginning of the Anthropocene (Swindles et al. 2015; Waters and Turner 2022). A similar pattern can be assumed for the other lakes that were not dated as far back in time. Concentrations before that, i.e., in the deepest layers of these cores (Fig. 3 and Online Resource Figs. S15–S17), are rather constant over a longer period of time and may be considered local background concentrations.

The first half of the twentieth century is dominated by three major world crises: the First World War (1914–1918), the Great (economic) Depression (1929–1936), and the Second World War (1939–1945) that had substantial influence on production and, thus, pollutant emissions (Niedertscheider et al. 2014). However, no obvious indications of these events were found. TE concentrations started to increase anywhere between the 1940s to the 1970s. The earlier increases could be attributed to war efforts, while later ones could be because of post-war recovery. These periods coincide with the “Great Acceleration,” a time of rapidly increasing, global anthropogenic impacts on the whole planet that is set to approx. 1950 (Swindles et al. 2015). It is marked by a rapid increase of spheroidal carbonaceous particles (SCPs) in stratigraphic records around the globe which are distinctly formed during burning of fossil fuels in energy production and heavy industry.

Starting from the 1960s, there is a remarkable general decline of TE concentrations. One would be inclined to accredit these reductions to policies and measures undertaken in the GDR to improve air quality. But the government failed to implement any substantial measures up to its demise in 1989, except for a reduction in fly ash of about 16% in the last 9 years (Buck 1996). Contrary to this, West Germany established its first legislation to control air pollution in 1964 (TA Luft 1964), followed 10 years later by 2 further novel legislature measures (BImSchG 1974; BImSchV No. 1 1974) regulating emissions of firing systems among others. A growing sense for the environment and its necessary protection essentially characterized this time period. These developments coincide nicely with the turning point in TE concentrations in the 1960s and the slightly stronger decrease in the 1970s, suggesting that the patterns in the cores are indicative of the atmospheric burden coming from West Germany instead of that from the GDR. In support of this theory, Pacyna (1984) identifies the Benelux countries and the western part of West Germany as a major emission area in Europe for that time, with emitted amounts of TEs that are approx. 3–26 times higher than those from the GDR. Additionally, the northern half of the former GDR, where the lakes are situated, harbored no greater industry or energy plants which could have served as local sources (Hornych and Schwartz 2009). Accordingly, the districts with the highest recorded dust emissions are in the south: Berlin (East), Cottbus, Halle, and Leipzig (Buck 1996). And finally, prevailing winds in the territory of the northern GDR are directed to the northeast (i.e., Westerlies), transporting air masses predominantly from the southwest to the lakes (Buck 1996; Traup and Kruse 1996; Bürger 2003). In this way, LRAT of air emissions from the southern GDR are rather directed to, e.g., Poland (Buck 1996), than to the lakes in the north of the GDR. Gunten et al. (1997) experienced the same sharp decline in lake sediment cores from Lake Zurich and ascribed this to environmental protection measures in Switzerland as the major cause as well.

The years afterwards (1980–2015) are characterized by a steady decline in TE concentrations to levels slowly approaching those before 1925 (Fig. 2). For the years 1990 until present, this is supported by national monitoring data of air emissions in Germany (UBA 2020), as well as bioaccumulation data of the national moss surveys (Schröder and Nickel 2019), and is accredited mostly to ash and SO_2_ reduction measures which are part of the legislative and technology measures to reduce air pollutants on an European level since the 1970s (Turnock et al. 2016). It remains to be seen, however, if those pre-industrial levels are reached in the future. Soils have been accumulating TEs for a very long time and are slowly releasing them, as several studies have shown (Yang et al. 2002; Bacardit and Camarero 2010; Catalan 2015), so that fluxes to the lakes can remain positive despite emission reductions.

The peak in ca. 2005 is reflected in the moss monitoring (Schröder and Nickel 2019). An increase in metal concentrations between 2000 and 2005 was also found in, e.g., Finland, Austria, and Switzerland, and is attributed to a chromium mine located on the Kola Peninsula (Kratz and Schröder 2010). Emissions of this mine could have been transported southwards to the lake area by temporary occurring weather systems.

As for local peculiarities, the resemblance in profiles between lakes FH, BL, SL, and CR is probably due to the geographic vicinity of the lakes to each other, and the hydraulic connections between them. Lakes ST and WM show the highest TE concentrations of all studied lakes. This might be in correlation to the high amount of forest in their catchment areas. Tree canopies are known to gather particles and pollutants from air, thereby increasing the input to the underlying soil (forest filter effect; Horstmann and McLachlan 1998; Nizzetto et al. 2006). The extraordinarily high Cu concentrations present in lake ST since 1966 and declining since 1981 could be associated with the adjacent power plant (Kernkraftwerk Rheinsberg) that was operated between 1966 and 1990 (Koschel and Adams 2003): It used water from the neighboring Lake Nehmitz as coolant. The warm waste water, however, was expelled into lake ST (UBA 2004) leading to an increase in average water temperature (O’Reilly et al. 2015). Presumably, the water could have carried Cu from the external cooling system through which it was transferred, as was observed in other power plant effluents (Wright and Zamudal 1991; Friedlander et al. 1996; Bojakowska and Krasuska 2014).

Contrary to expectation, no indication of traffic emissions, i.e., through Pb concentrations mirroring the use of leaded gasoline, was found. Instead, the TE profiles of the lakes mirror emissions from industrial processes (Heinrichs 1993; Wessels et al. 1995). Possibly, emitted Pb did not reach the lakes or it had too little effect on the overall Pb concentration, so that it was masked by significantly higher industrial depositions. In Switzerland, cores from Lake Zurich showed rising Pb concentrations long before the introduction of leaded fuel (Gunten et al. 1997), and in Lake Brêt, the Pb profile roughly followed that of other studied elements, i.e., Cu and Hg (Thevenon et al. 2013). Both observations, which are present in the current study as well, were ascribed to an overall metal pollution from industrial activity as the major source.

### Organochlorine pesticides

Of the DDT group of analytes, only TPs DDD and DDE but no traces of the parent compound DDT were detected. The period with the strongest increases and peaks of concentrations was between ca. 1945 and ca. 1975, excluding lake TF featuring the main peak in ca. 1985. Concentrations of ΣDDX peaked between 30 and 380 µg·kg^−1^ (median 225 µg·kg^−1^). These are, in fact, comparable to 4 of the 5 Canadian lakes examined by Kurek et al. (2019), which experienced direct aerial spray input of DDT in the 1950s–1970s, with maxima between ca. 130 and ca. 575 µg·kg^−1^.

MacDonald et al. (2000) developed consensus-based sediment quality guidelines (SEQs) for 28 chemicals of concern in freshwater ecosystems. Among them were a threshold effect concentration (TEC) and a probable effect concentration (PEC; not to be mixed up with predicted environmental concentration) for each analyte of concern. The TEC is intended to identify concentrations below which detrimental effects on sediment-dwelling organisms are not expected. The PEC on the other hand should indicate concentrations above which harmful effects on such organisms are expected to occur frequently. In the cases of ΣDDD and ΣDDE, TECs were 4.88 and 3.16 µg·kg^−1^, respectively, and PECs were 28 and 31.3 µg·kg^−1^, respectively. The latter were generally exceeded during the mentioned period of highest concentrations, by 2 to as much as 10 times in the case of ΣDDD, and by almost 3 times in the case of ΣDDE. Thus, detrimental effects for benthic fauna during the period of usage were very likely. Concentrations near the surface of the lake sediment were mostly < 10 µg·kg^−1^. They were in the same range as other surface sediment samples from anthropogenically influenced lakes. Similar concentrations as those found in our study were also observed in Lake Victoria in Uganda (mean ΣDDX 4.24 µg·kg^−1^; Wasswa et al. 2011), Chinese Lakes Taihu (ca. 3.7–13.6 µg·kg^−1^; Zhao et al. 2017), and Songyang (0.4–18.4 µg·kg^−1^; Gong et al. 2021). In contrast, concentrations of DDD and DDE of some lakes (AR, BL, OR, PL) were similar to those found in remote alpine lakes of Switzerland (< LOD–4.75 µg·kg^−1^; Poma et al. 2017). Concentrations in these four lakes are well below the TECs, indicating no acute threat to epibenthic fauna. Nevertheless, sublethal and chronic effects might still occur (Chattopadhyay and Chattopadhyay 2015; Marziali et al. 2017; Iliff et al. 2019).

For further considerations, it is important to establish if the concentrations of DDD and DDE as well as the ratio between them have significantly changed over the decades since their deposition. First of all, a transformation of DDD to DDE or vice versa in the sediments is rather unlikely. Although both transformation paths have been reported (Purnomo et al. 2008, 2011), they were catalyzed by brown-rot fungi that require aerobic conditions (Kim and Singh 2000), which does not apply here. Microbial degradation of DDD and DDE under anaerobic conditions has been reported. For DDE, the most probable path of transformation is to DDMU (1-chloro-2,2-bis-(p-chlorophenyl)ethylene) as the next following product via reductive dechlorination (3 times faster than DDD; Quensen et al. 2001; Schulze et al. 2003; Yu et al. 2011). The dominant anaerobic transformation pathway of DDT results in both DDD and DDMS (1-chloro-2,2-bis-(p-chlorophenyl)ethane), as shown, e.g., by Heim et al. (2005) and Yu et al. (2011). Apart from DDD and DDE, other TPs were not analyzed in the current study. Coincidentally, Heim et al. (2005) studied a sediment core from Teltow canal in Berlin, which was severely exposed to DDT and lindane in the former GDR, albeit by a chemical factory (VEB Berlin Chemie). They found DDMS as the second most abundant TP after DDD, which showed at least 2 times higher concentrations. The lowest part of their core had been deposited 7 years before the sampling. Concentrations of DDD were still 44 times above those of DDT and 15 times above DDE. Therefore, we assume that even if degradation of the primary TPs has occurred, the overall ratio of DDD to DDE has not been changed fundamentally up to this point.

For these reasons, we assume two distinctive scenarios: In the first, DDT formulation directly reaches the lake sediment, e.g., because of drift during application on fields and forests (Frank et al. 1994; Craig et al. 1998; Matthews 2014). In this scenario, mainly DDT is deposited, and while minor parts may be transformed to DDE during deposition, most of the DDT is afterwards transformed to DDD anaerobically. In the second scenario, DDT reaches the sediment indirectly via erosion of topsoil some time after agricultural or forest application. The proportion of DDT in the eroded material will be higher at first, but with ongoing dwell time and, thus, transformation in the mostly aerobic soil, DDE concentrations will increase until they finally surpass those of DDT, if no additional DDT formulation is applied (lakes CR, OR, and SL; Dimond and Owen 1996). Depending on the capacity of reservoirs capable of storing DDT surrounding the lakes, a more or less constant leakage input is imaginable, leading to steadily low concentrations in the profiles (lake TF). Such a behavior of persistent chlorinated pollutants was observed in boreal forests of Norway by Bergknut et al. (2011). In reality, these two scenarios will most probably occur coincidentally to different extents in different points of time.

Furthermore, input of DDX into the lakes or their surrounding reservoirs via LRAT is possible and has been reported (Juracek and Mau 2003). However, contributions through this pathway should be minor in regard to the extent of direct depositions that took place in the examined area.

Silva et al. (2019) evaluated the distribution of 76 pesticides in agricultural topsoil samples from across the EU in 2015. Frequently found compounds were DDT and its TPs. They were second only to the herbicide glyphosate which was applied much more recently. Accordingly, the most common TP was 4,4’-DDE with median and maximum of 20 and 310 µg·kg^−1^, respectively, while for 4,4’-DDD, it was 10 and 40 µg·kg^−1^. In the current study, concentrations in the youngest layers dating back to 2010–2015 were between < LOD and 15.71 for 4,4’-DDE and < LOD and 22.07 µg·kg^−1^ for 4,4’-DDD. Camenzuli et al. (2016) evaluated data from 73 peer-reviewed articles and calculated median concentrations for several legacy pesticides of agricultural and background soils from all over the world. The results were divided into two distinct periods: 1993–2002 and 2003–2012. Values of 4,4’-DDT declined from 16.22 to 8.71 µg·kg^−1^ in agricultural soils and from 1.23 to 0.91 µg·kg^−1^ in background soils. Similar values and trends were shown for 4,4’-DDE: 12.02 to 7.76 µg·kg^−1^ and 0.98 to 0.51 µg·kg^−1^ in agricultural and background soils, respectively. In the present study, 4,4’-DDD which is assumed to represent its DDT counterpart, is found at a median concentration of 7.66 µg·kg^−1^ in the first period and at < LOD in the second. 4,4’-DDE stays more or less the same (7.23 and 8.23 µg·kg^−1^), at concentrations comparable to those from agricultural soils in the second period from Camenzuli’s study. The predominant TP in other studies concerning OCP concentrations in sediments was DDD (Muir et al. 1995; Hendy and Peake 1996; Pereira et al. 1996; Hoke et al. 1997; Eggen and Majcherczyk 2006; Götz et al. 2007; Thevenon et al. 2013), and in two of them examining river sediments, almost all DDT was converted (Schwarzbauer et al. 2001; Heim et al. 2005). On the other hand, there are also many studies in which DDE was the predominant TP (e.g., Rawn et al. 2001; Juracek and Mau 2003; Franců et al. 2010; Bettinetti et al. 2011). Franců et al. (2010) and Evenset et al. (2007) attributed this to input of mainly strongly weathered soil material (i.e., from agriculture), and little direct input of DDT into the lake.

#### Historical context

The insecticidal feature of DDT was discovered in 1939 and came to widespread use in agriculture following World War 2 (Mellanby 1992 ex Jürgens et al. 2016; Ricking and Schwarzbauer 2012). In the 1950s and 1960s, the application of DDT increased dramatically. But rising awareness of environmental issues in the 1960s also affected the agricultural sector. By that time, DDT had grown into a model compound in ecotoxicological studies, whose unfavorable results led to waning enthusiasm for the pesticide (Mellanby 1992 ex Jürgens et al. 2016). At the end of the 1960s, the GDR government found itself constrained to react to the global wave of DDT bans, which endangered its own food and crop exports. As a result, a stepwise substitution plan was adopted: From 1971 until 1976, more and more DDT bans for the most application intensive crops were installed, visible in decreasing amounts of distributed DDT in the GDR from ca. 280 Mg·a^−1^ in 1972 to ca. 20 Mg·a^−1^ in 1977 (Heinisch et al. 1993). Handout and assumed applications stayed low until 1983/1984, when the massive spread of the nun moth (*Lymantria monacha*) and associated pests (Riek et al. 2021) in forests of the northern GDR forced the government to divert from the substitution plan. Unofficially, 260,000 ha of the 600,000 ha of infested stands were aviochemically treated with DDT/lindane formulations. After that, handout of DDT dropped back to the previous level of 1970 and quickly declined further, finally coming to an end shortly before Germany’s reunification a few years later (Heinisch et al. 1993).

These developments are reflected in the 4,4’-DDD concentrations of the single profiles (Fig. 4). The increase after World War 2 is present in all studied lakes where the core covers this time period (except lake AR which only shows one increase from 1970 and subsequent decrease after 1975). The main application period in the 1950s and 1960s is especially prominent in lakes FH, BL, SL, OR, and even in the forest lake ST. Probably as a result of the stepwise ban, concentrations are lower at the beginning of the 1970s in lakes FH, BL and OR, while the latter is demonstrating a gradual decline. In some areas, local farmers or foresters might have resorted to applying higher rates than usual in order to dispose of their remainders of the soon banned pesticides. This in turn could have led to high concentrations in lakes CR, PL, and AR in the 1970s, and through them to the peak in Fig. 2. In other studies of lake sediments, the main peak of DDX concentration was also found in the mid-1960s to mid-1970s in European countries, e.g., GB (Fox et al. 2001), Germany (Götz et al. 2007), Italy (Bettinetti et al. 2011), and Norway (Evenset et al. 2007), but also in several lakes throughout Canada (Rawn et al. 2001; Kurek et al. 2019) and the USA (van Metre and Mahler 2005). The secret operation against the nun moth in 1983/1984 is impressively visible in lake TF, and is indicated to a lesser extent in lakes BL (ca. 1985) and OR (ca. 1986).

Lakes FH, SL, TF, and OR demonstrate a slight increase in DDD and DDE concentrations after the year 2000. Sabatier et al. (2014) described the same phenomenon in a sediment core from a lake situated downhill of a vineyard in France in the 1990s. According to their theory, legacy pesticides (DDT among others) were remobilized from the soil by increased erosion, which in turn was caused by the application of the herbicide glyphosate, as it leads to a reduction in canopy above the soils, leaving them vulnerable to erosion. A similar effect could be imaginable here.

#### Regional traits

In the days of the GDR, lakes FH, BL, SL, CR, OR, and TF belonged to one single district (Neubrandenburg), and lakes ST and WM to another (Potsdam). Guidelines of these distinct administrations could play a role in existing similarities within each of these two groups. Still, circumstances on a much more local scale can contribute largely. The group of lakes around lake FH (FH, BL, SL, CR) are close in terms of distance and connection. Nevertheless, they did not show four almost identical profiles but can be divided into pairs: Lakes FH and BL are quite similar, as are lakes SL and CR. Concentrations of OCPs of the four lakes follow this order: FH ≈ BL < SL < CR. Considering the absolute amounts of agriculture and forest area surrounding these lakes in their hydrological catchment area, there is a similar pattern: FH < BL < SL < CR. The influence of forest area in the vicinity of the lakes needs to be stressed not only for TEs but also for OCPs, although in this case, the contribution of LRAT in conjunction with the forest filter effect was probably minor in comparison to the effect of the direct aerial pesticide applications in the GDR. In Switzerland, Herzig et al. (2019) analyzed the DDX pollution load (among others) in lichens collected in 1995 and 2014. They found significant declines between 56 and 84% and credited this to POP emission regulations in Switzerland as part of central Europe, leading to decreasing direct inputs.

Another important point is the high capability of forest soils to store hydrophobic substances (Holoubek et al. 2009; Riek et al. 2021). This can lead to reemissions long after the last application. Aichner et al. (2013) examined the distribution of POPs including DDT and its TPs in forest soils throughout Germany. They found that concentrations in eastern Germany were much higher than in western Germany (equivalent to the former areas of the GDR and West Germany, respectively). For the region of the sampled lakes, ca. 160–440 µg·kg^−1^ for 4,4’-DDT, ca. 40–110 µg·kg^−1^ for 4,4’-DDE, and ca. 4–11 µg·kg^−1^ for 4,4’-DDD were measured, which was at least one order of magnitude higher than in western Germany. The authors state the severe forest treatment in the former GDR as reason for this. The concentrations in our study are within or below this range.

#### DDX congener ratios

The mostly unchanging 4,4’-DDD/2,4’-DDD ratios suggest a consistent quality of applied DDT formulations.

Ratios of 4,4’-DDD/4,4’-DDE are in accordance with the two scenarios proposed before. During the officially recorded time range of direct input (ca. 1945–1970), most values are fairly above 1. The decline of 4,4’-DDD/4,4’-DDE ratios that started in the late 1950s could indicate a rising importance of DDE input through erosion after aerobic DDT degradation. By 1985, many lakes have approached values near 1.

#### HCH

Unlike DDT, for which the ban in the 1970s led to rapidly declining applications, HCH remained an important plant protection product in the GDR until the state’s end in 1990. Though a negative trend in the number of licensed products occurred, HCH was produced and used intensively in many fields of the GDR (e.g., agriculture, forest, hygiene, material protection, private), which led to highly contaminated agricultural soils (Heinisch and Klein 1992). Nevertheless, no HCH was found in the current study. This is probably due to its physico-chemical properties (Camenzuli et al. 2016) which influence its overall persistence. This is in line with the study of Bidleman et al. (2021) who examined the dissipation of HCH in Lake Superior, Canada. Between 1986 and 2016, concentrations declined by more than 90%. The authors identified volatilization as main removal process, followed by (hydrolytic and microbial) degradation and outflow through the draining river. Sedimentation was deemed minor.

## Conclusion

In this study, 10 sediment profiles from 10 lakes in northeastern Germany (today’s Mecklenburg-Western Pomerania and Brandenburg) covering an area of ca. 40,000 km^2^ were analyzed for elemental and pesticide concentrations. The generated time series are the first of their kind for the examined area. As intended, they provided evidence of anthropogenic impact in the covered area. Indicators of the Industrial Revolution, the post-war recovery, and the beginning of environmental legislation measures in the 1960s and 1970s were found.

TE concentrations represented air emissions of inorganic pollutants from industrialized areas in western Germany and Europe, indicating industrial activity and policy making with long-range impact. OCP concentrations were consistent with local and regional use in agriculture and forestry, recording periods and single events of pesticide applications and their intensity with rather short-range impact. OCPs also provide evidence for anthropogenic developments and activities hidden from the general public in the former GDR. Concentrations of OCPs in deeper sediment layers exceeded toxic limits, while those on the surface of the lake bottom did not. Both elemental and OCP concentrations were heavily influenced by short- and long-range human activities and respective political and legal measures implemented through time.

Sedimentary TE profiles are in accordance with other forms of long-term monitoring, i.e., the European moss monitoring survey (UBA 2019) and are suited to complement and validate these and others, e.g., the Long-Term Ecological Research Network (LTER) and the Global Lake Ecological Observatory Network (GLEON). Furthermore, sedimentary TE profiles can be used as a relatively cheap and easy dating reference point for northeastern Germany for future paleolimnological works, i.e., as age models solely based on elemental patterns.

Complementing the results from this study with further data from lakes in southeastern Germany and from western Germany would be highly desirable to test for environmental impacts by short- and long-range human activities through time. This would also enable comparison between agricultural and industrial areas. Additional soil samples in combination with satellite observations of the different land-use areas around the lakes could supplement our theories and findings and facilitate inferences about the more recent situation. Lastly, if the analytical portfolio is extended with additional TPs of OCPs, conclusions about the state of degradation could be made with more confidence. Overall, the current study proves the suitability of dated lacustrine sediments to reliably reconstruct anthropogenic development and disturbance events in the past, as well as retroactively assess the success of legislative regulatory measures.

## Supplementary Information

Below is the link to the electronic supplementary material.

Supplementary file1 (PDF 2.12 MB)

## Data Availability

Data for this study were published open access (Simon et al. 2023). Additional figures and tables mentioned in the text are available as part of the Online Resource on the article’s webpage (Supplementary file 1; PDF).
