# Supplementary material for: Dissent in the sediment? Lake sediments as archives of short- and long-range impact of anthropogenic activities in northeastern Germany
Source: Environ Sci Pollut Res Int. 2023 Jul 3;30(36):85867–88. doi: 10.1007/s11356-023-28210-8 (PMC10404210; doi:10.1007/s11356-023-28210-8)
Supplement: Supplementary file 1 — Supplementary file1 (PDF 2.12 MB) [file 11356_2023_28210_MOESM1_ESM.pdf]

Electronic supplementary material to article

Dissent in the sediment? Lake sediments as archives of short- and long-range impact of anthropogenic activities in northeastern Germany

(https://doi.org/10.1007/s11356-023-28210-8)

Journal
Environmental Science and Pollution Research (ESPR)
(http://www.springer.com/environment/journal/11356)

Authors

Marcel Pierre Simon	https://orcid.org/0000-0003-3007-699X
Marlene Schatz	
Leonard Böhm	https://orcid.org/0000-0002-3435-5956
István Papp	https://orcid.org/0000-0002-0006-9919
Hans-Peter Grossart	https://orcid.org/0000-0002-9141-0325
Thorbjørn Joest Andersen	https://orcid.org/0000-0001-5032-9945
Miklós Bálint	https://orcid.org/0000-0003-0499-8536
Rolf-Alexander Düring	https://orcid.org/0000-0002-4329-1042

Corresponding author
Marcel Pierre Simon (Marcel.P.Simon@umwelt.uni-giessen.de)
Institute of Soil Science and Soil Conservation, Research Centre for BioSystems, Land Use and Nutrition (iFZ), Justus Liebig University Giessen, Heinrich-Buff-Ring 26-32, 35392 Giessen, Germany

Table of contents

		Page
Fig S1	Similarities between cores from lakes BL and FH	2
Fig S2	Alignment between cores from lake AR	2
Fig S3	Re-evaluated age models of lakes BL and TF	3
Table S1	Internal standard mix	4
Table S2	MAE-AR program	4
Table S3	List of ions and retention times (RTs)	4
Table S4	Overview of data points comprising sum of trace elements in Fig. 1	5
Table S5	Overview of data points comprising sum of DDX in Fig. 1	5
Fig S4	Isotope activities of the lake cores	6
Figs S5-S14	Elemental profiles of each lake	7–16
Figs S15-S17	Elemental data that precedes 1900: lakes SL, CR, PL, ST, and, WM	17–19
	References	19

Data repository
https://doi.org/10.1594/PANGAEA.951049

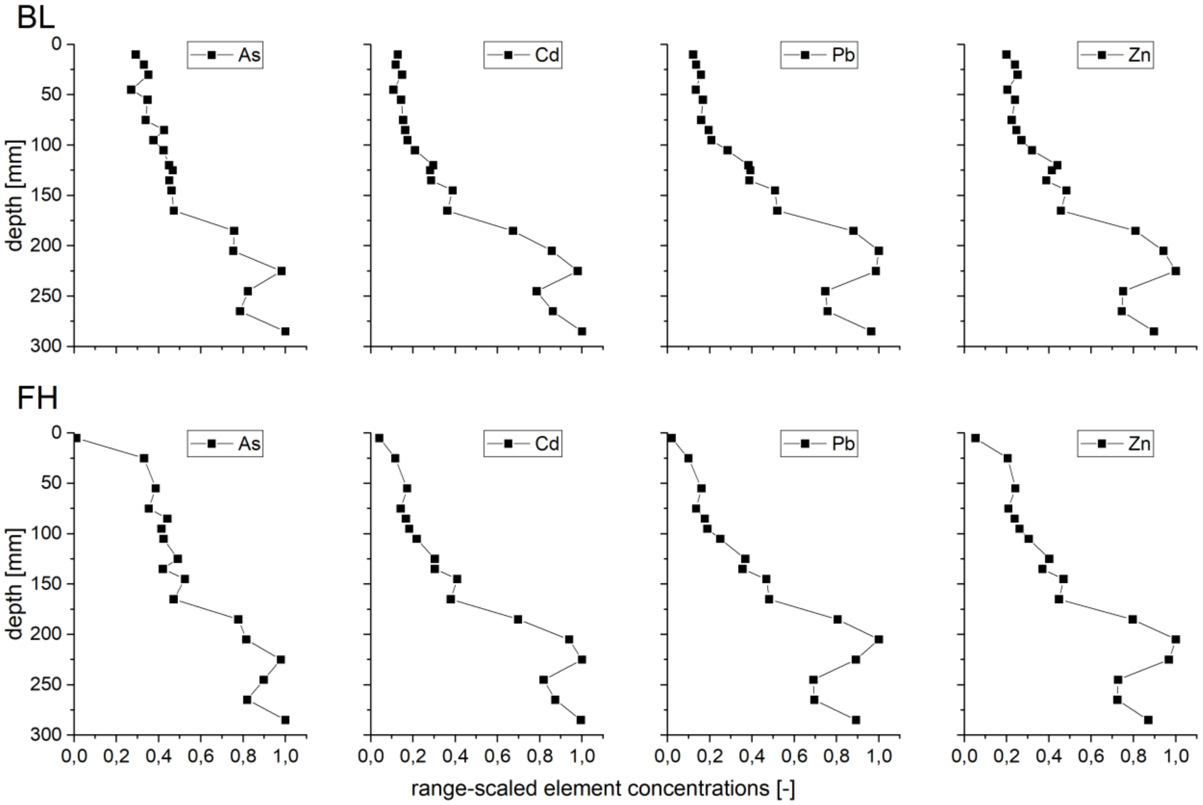

Fig. S1 Normalized concentration profiles of trace elements (TEs) As, Cd, Pb and Zn of cores from Lakes Breiter Luzin (BL) and Feldberger Haussee (FH) demonstrating similarities

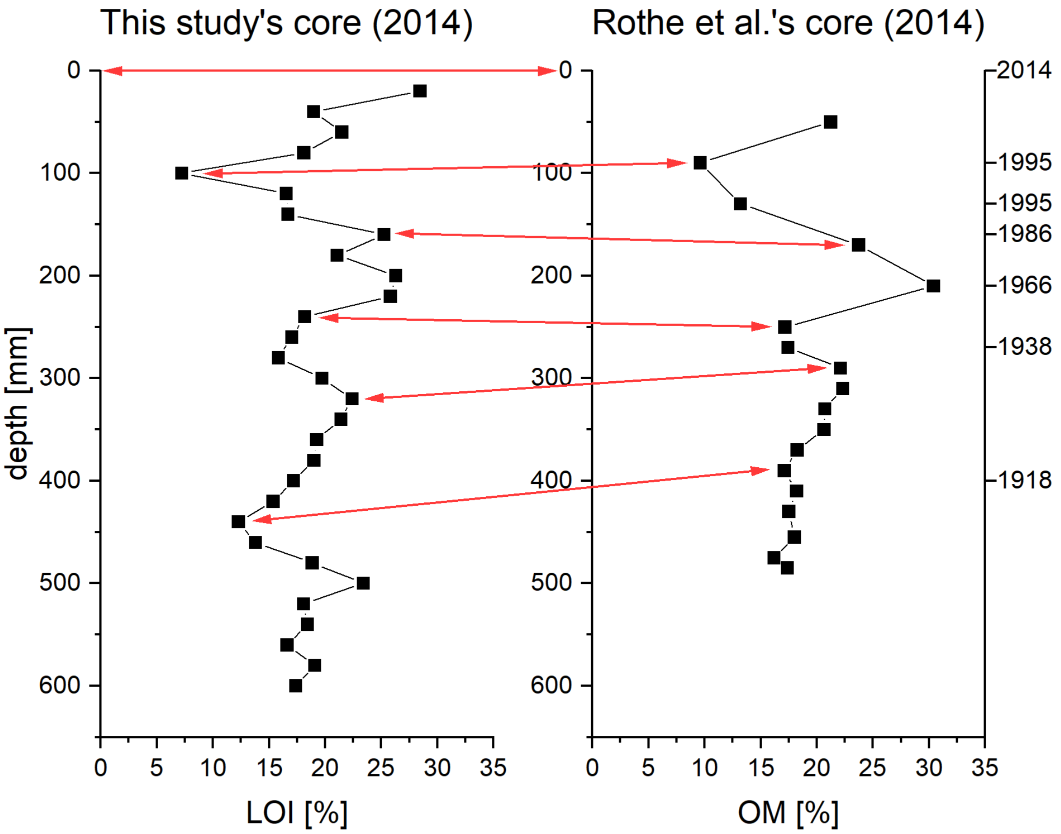

Fig. S2 Alignment of cores from Lake Arendsee (AR) from this study and of Rothe et al. (2015) via content of organic matter. LOI = loss on ignition, OM = organic matter

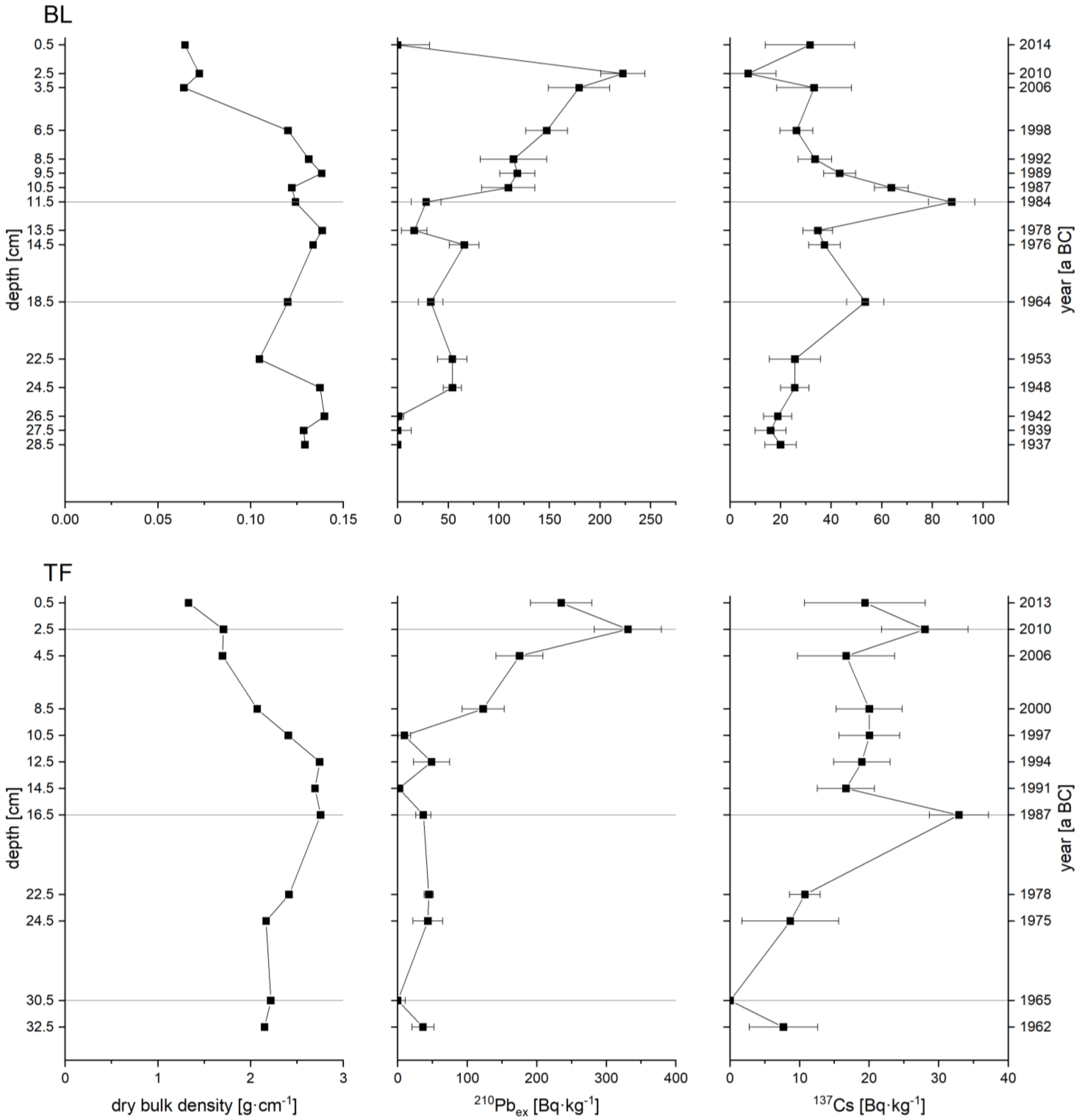

Fig. S3 Re-evaluated age models of cores from Lakes Breiter Luzin (BL) and Tiefwaren (TF)

Table S1 Microwave program for MAE-AR extraction

	Power (W)	Limit Temp. (°C)	Hold time (min)
Step 1	250	–	1
Step 2	250	–	15
Step 3	500	–	10
Ventilation	–	–	30

Table S2 Composition of the applied internal standard mix (stock solution)

Component	Concentration [$\mu g \cdot mL^{-1}$]	Final concentration in sample [$ng \cdot mL^{-1}$]
4,4'-DDE-D_8	5	1
4,4'-DDD-D_8	10	2
^{13}C-2,4'-DDT	15	3
^{13}C-4,4'-DDT	15	3
α-HCH-D_6	15	3

Table S3 Retention times and ions used to identify and quantify the analytes. RT = Retention time, IS = Internal standard. α-HCH-D_6 was used to correct all HCH congeners. 4,4'-DDE-D_8 was IS for both DDE congeners, and 4,4'-DDD-D_8 for both DDD congeners. The ^{13}C substituted IS were used to correct their respective unmarked counterparts

Name	Purpose	RT [min]	Quantifier [$m \cdot z^{-1}$]	Qualifier [$m \cdot z^{-1}$]
α-HCH-D_6	IS	21.15	224	185
α-HCH	Analyte	21.36	219	181
γ-HCH	Analyte	22.70	219	181
β-HCH	Analyte	23.92	219	181
δ-HCH	Analyte	24.98	219	181
2,4'-DDE	Analyte	29.02	246	318
4,4'-DDE-D_8	IS	30.37	254.2	326
4,4'-DDE	Analyte	30.46	246	318
2,4'-DDD	Analyte	30.82	235	165
^{13}C-2,4'-DDT	IS	32.00	247	177
2,4'-DDT	Analyte	32.01	235	165
4,4'-DDD-D_8	IS	32.38	243	173
4,4'-DDD	Analyte	32.50	235	165
^{13}C-4,4'-DDT	IS	33.68	247	177
4,4'-DDT	Analyte	33.68	235	165

Table S4 Number of data points included of each lake in the periods used to create the overview graph for *sum of trace elements* in Fig. 2

Period	Lake										No. of data points	No. of lakes included
	FH	BL	SL	CR	TF	OR	PL	AR	ST	WM		
2017-2013	1	1	0	1	0	2	1	0	1	0	7	6
2012-2008	1	2	1	1	2	1	1	1	1	1	12	10
2007-2003	0	1	1	1	2	1	1	1	1	1	10	9
2002-1998	1	1	1	0	1	2	0	2	0	1	9	7
1997-1993	1	1	0	1	2	2	1	1	1	0	10	8
1992-1988	2	2	1	0	1	3	1	1	0	1	12	8
1987-1983	2	2	1	1	2	2	1	1	0	0	12	8
1983-1978	2	3	0	0	1	1	0	2	1	1	11	7
1977-1973	1	1	1	1	0	2	1	1	0	1	9	8
1972-1968	1	1	1	1	1	1	1	1	0	1	9	9
1967-1963	1	1	0	0	1	1	1	1	1	1	8	8
1962-1958	1	1	1	0	1	1	1	1	0	1	8	8
1957-1953	1	1	0	1	1	1	0	1	0	1	7	7
1952-1948	1	1	1	0	0	0	1	1	0	1	6	6
1947-1943	0	0	0	0	0	1	1	2	1	1	6	5
1942-1938	1	1	1	1	0	0	1	1	0	1	7	7
1937-1933	1	1	1	0	0	0	0	1	0	1	5	5
1932-1928	0	0	0	0	0	0	1	1	0	1	3	3
1927-1923	0	0	0	1	0	0	0	2	1	1	5	4

Table S5 Number of data points included of each lake in the periods used to create the overview graph for *sum of DDX* in Fig. 2

Period	Lake										No. of data points	No. of lakes included
	FH	BL	SL	CR	TF	OR	PL	AR	ST	WM		
2017-2013	1	0	1	1	0	2	1	0	0	0	6	5
2012-2008	2	1	1	1	2	1	1	1	1	0	11	9
2007-2003	1	2	0	1	2	1	1	1	1	0	10	8
2002-1998	1	1	1	0	1	2	0	1	0	0	7	6
1997-1993	1	1	0	1	2	2	1	0	0	0	8	6
1992-1988	2	2	1	0	1	3	1	0	0	0	10	6
1987-1983	1	2	1	1	2	1	1	0	0	0	9	7
1983-1978	1	2	0	0	1	1	0	2	2	0	9	6
1977-1973	0	1	1	1	0	2	1	1	1	0	8	7
1972-1968	2	1	1	1	1	1	1	0	1	0	9	8
1967-1963	1	1	0	0	1	1	1	1	1	0	7	7
1962-1958	1	1	1	0	1	1	1	0	2	0	8	7
1957-1953	2	1	0	1	1	1	0	0	1	0	7	6
1952-1948	1	1	1	0	0	0	1	0	0	0	4	4
1947-1943	1	0	0	0	0	1	1	0	1	0	4	4
1942-1938	1	1	1	1	0	0	1	0	1	0	6	6
1937-1933	0	1	1	0	0	0	0	0	0	0	2	2
1932-1928	1	0	0	0	0	0	1	0	1	0	3	3
1927-1923	0	0	0	1	0	0	0	0	2	0	3	2

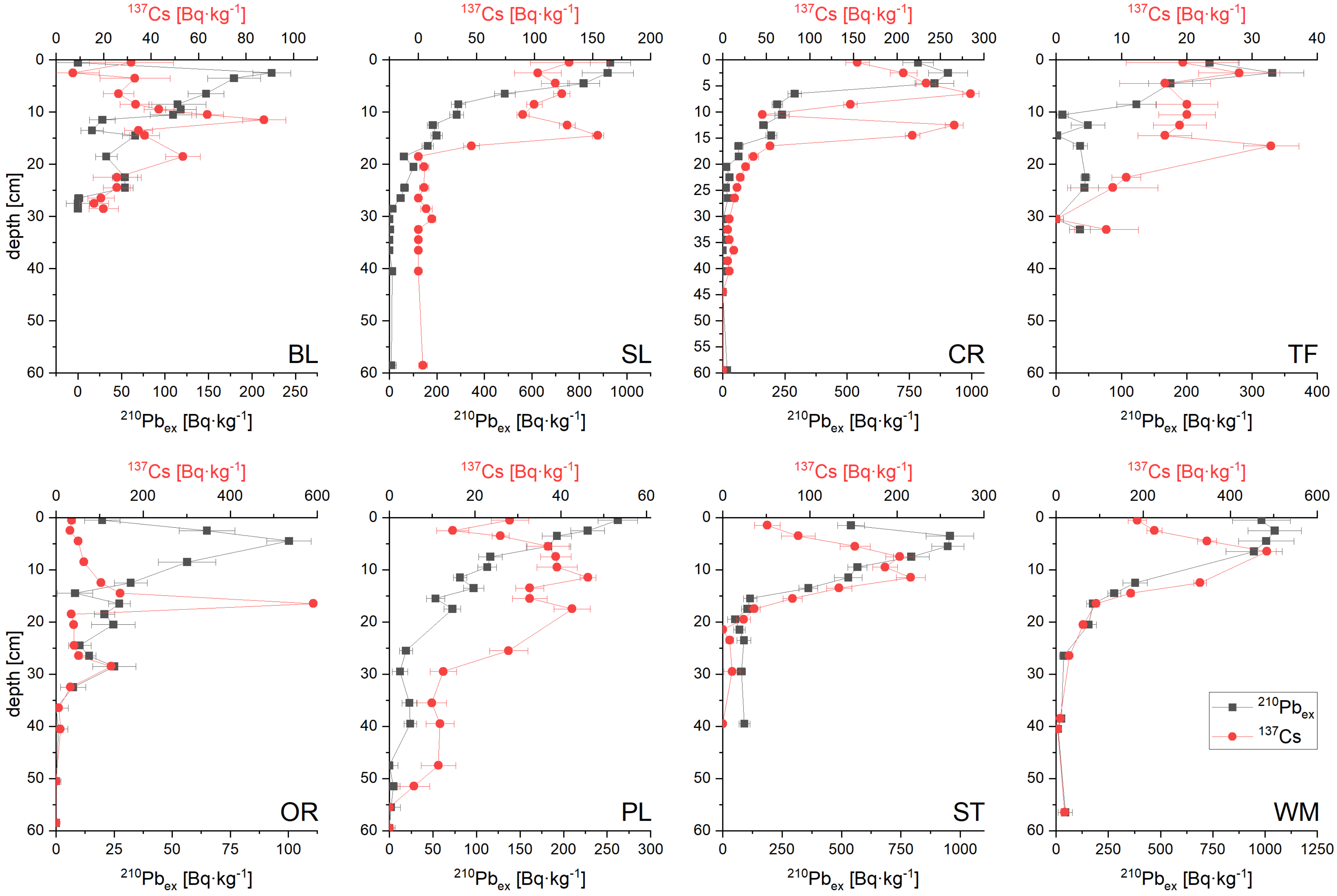

Fig. S4 Activities of isotopes ^{210}Pb (red) and ^{137}Cs (dark gray) per depth in each lake profile. Error bars depict standard deviation

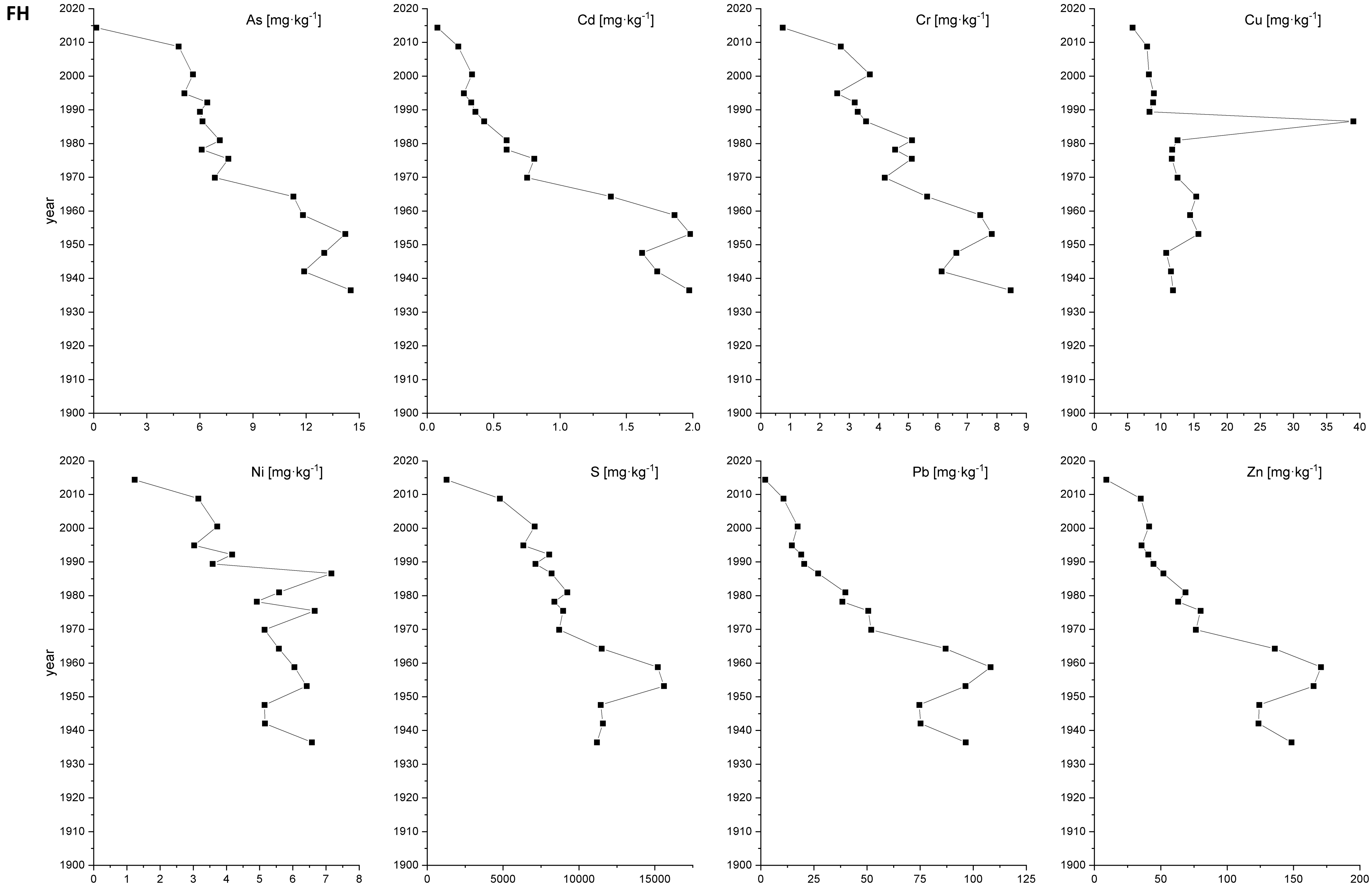

Fig. S5 TE concentrations of the core from Lake Feldberger Haussee (FH) in mg·kg^{-1}. Please note the differing x-axes

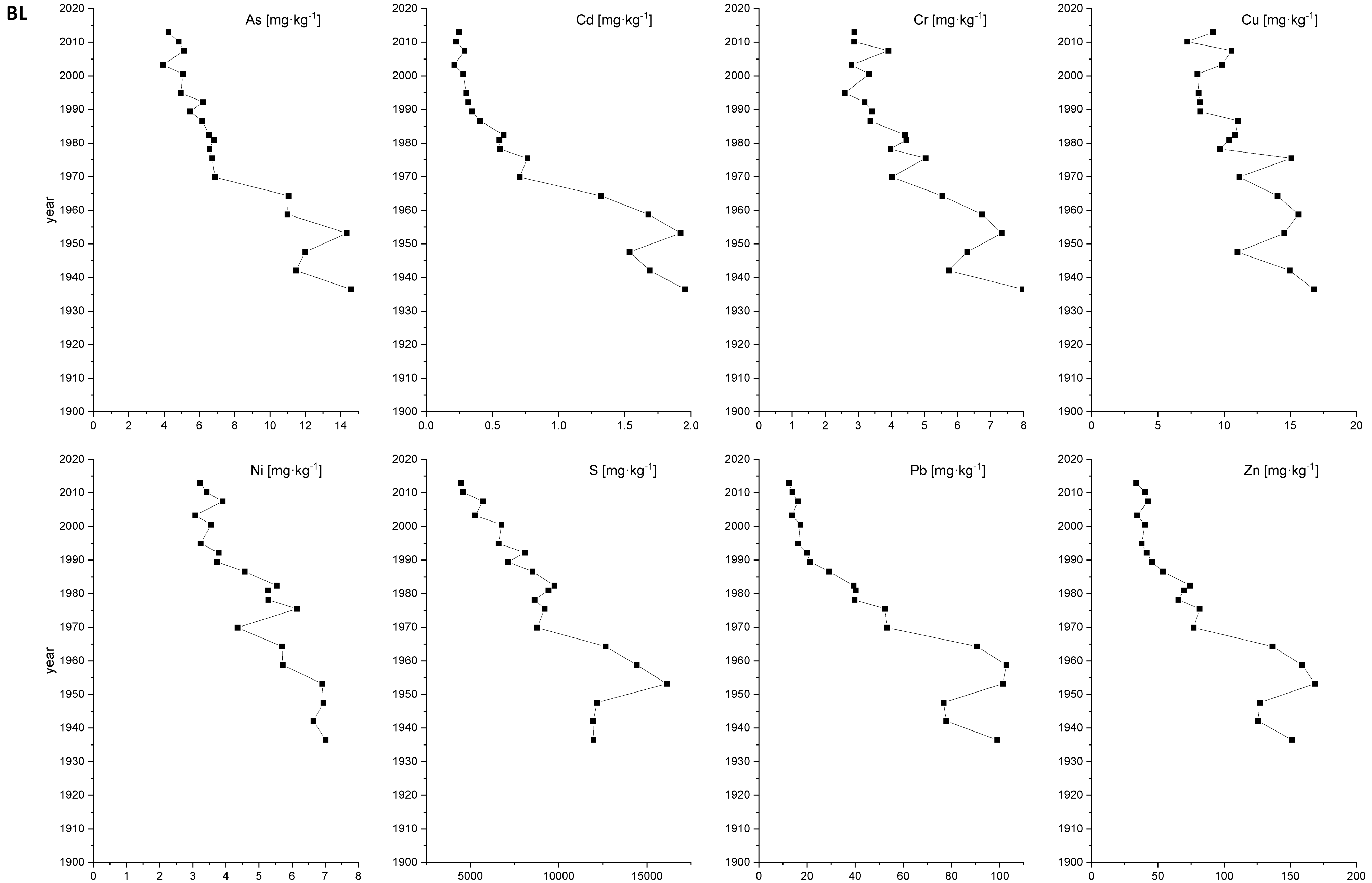

Fig. S6 TE concentrations of the core from Lake Breiter Luzin (BL) in mg·kg^{-1}. Please note the differing x-axes

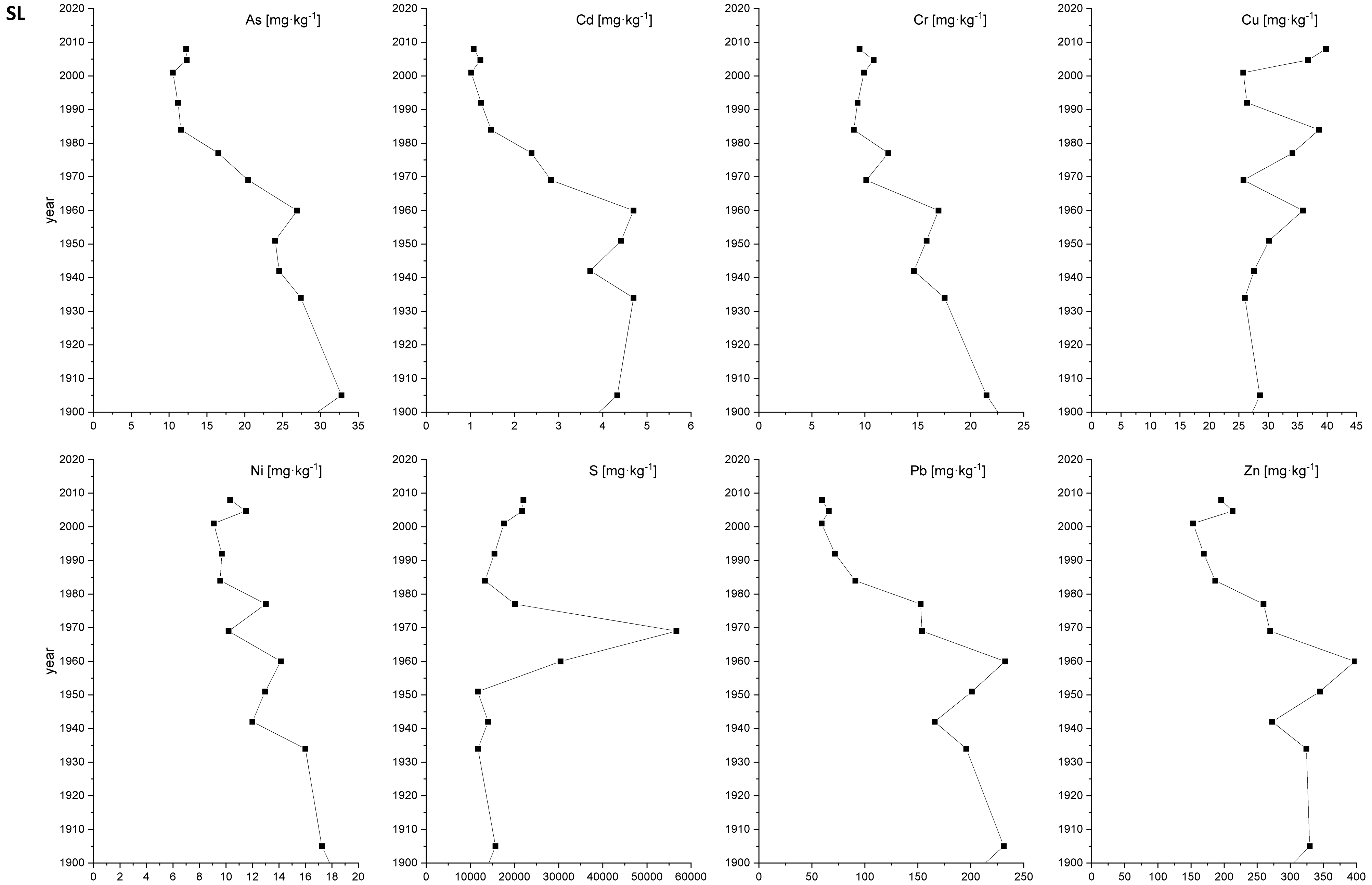

Fig. S7 TE concentrations of the core from Lake Schmaler Luzin (SL) in mg·kg^{-1}. Please note the differing x-axes

CR

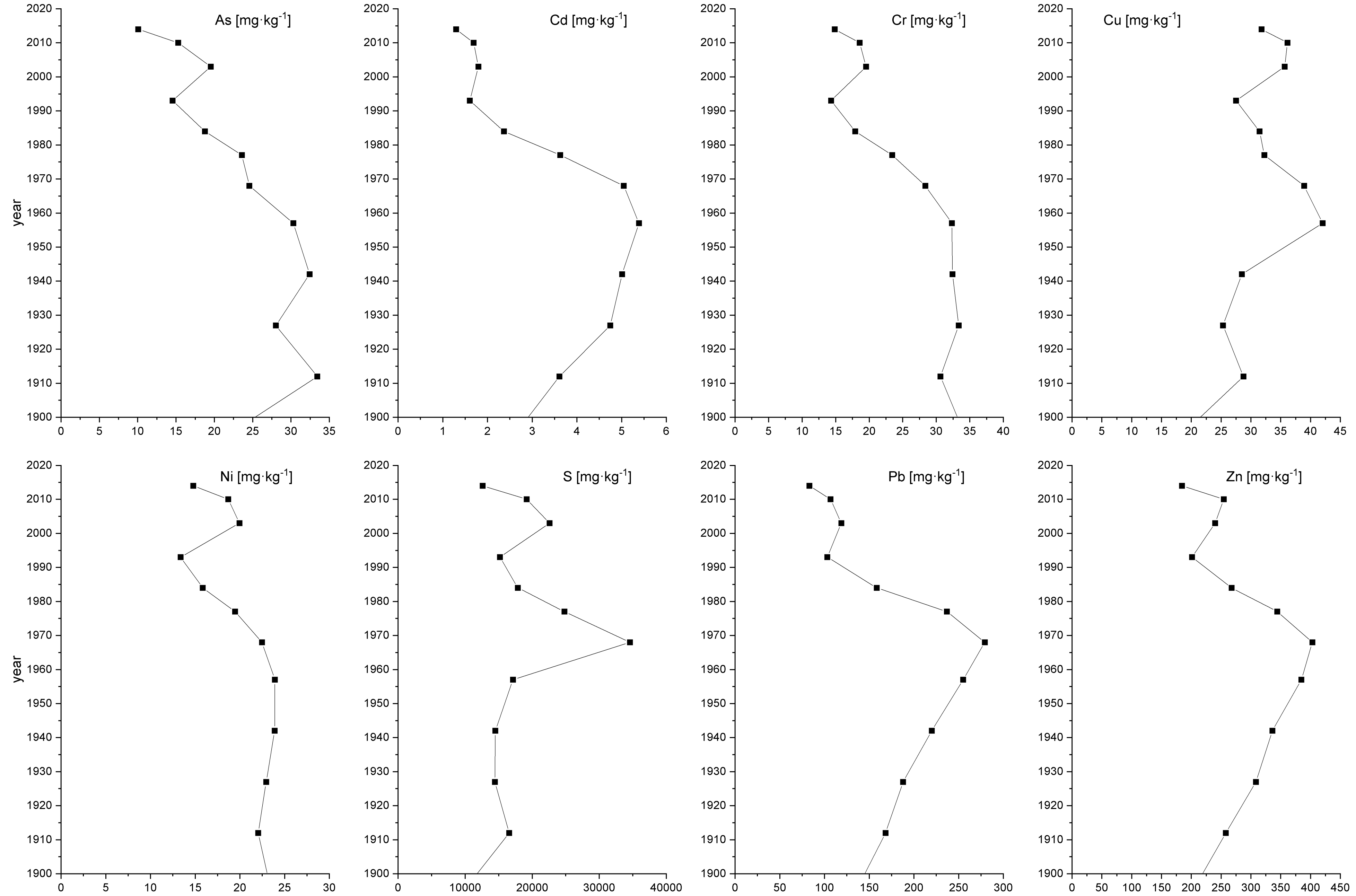

Fig. S8 TE concentrations of the core from Lake Carwitzer (CR) in mg·kg^{-1}. Please note the differing x-axes

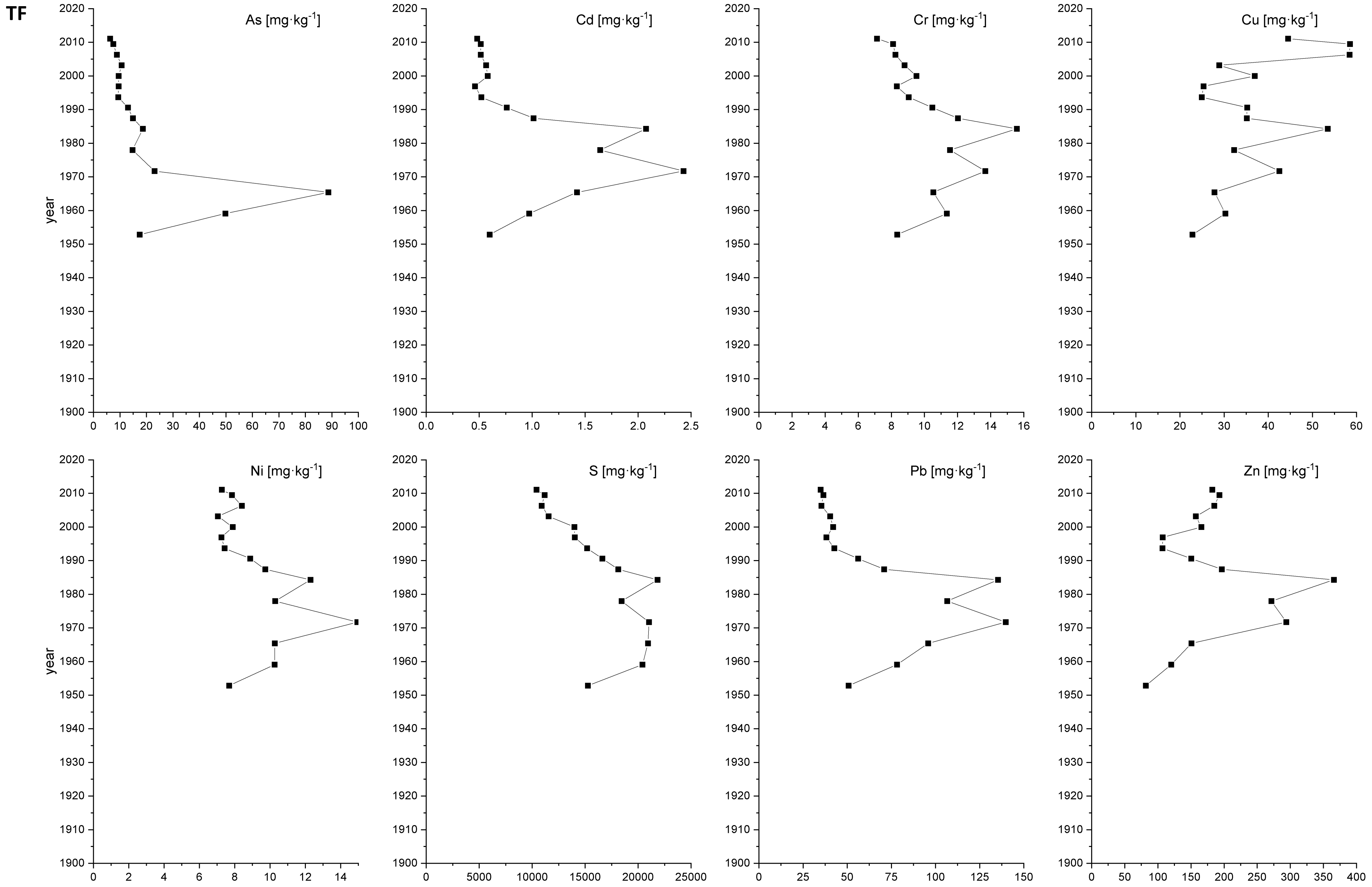

Fig. S9 TE concentrations of the core from Lake Tiefwaren (TF) in mg·kg^{-1}. Please note the differing x-axes

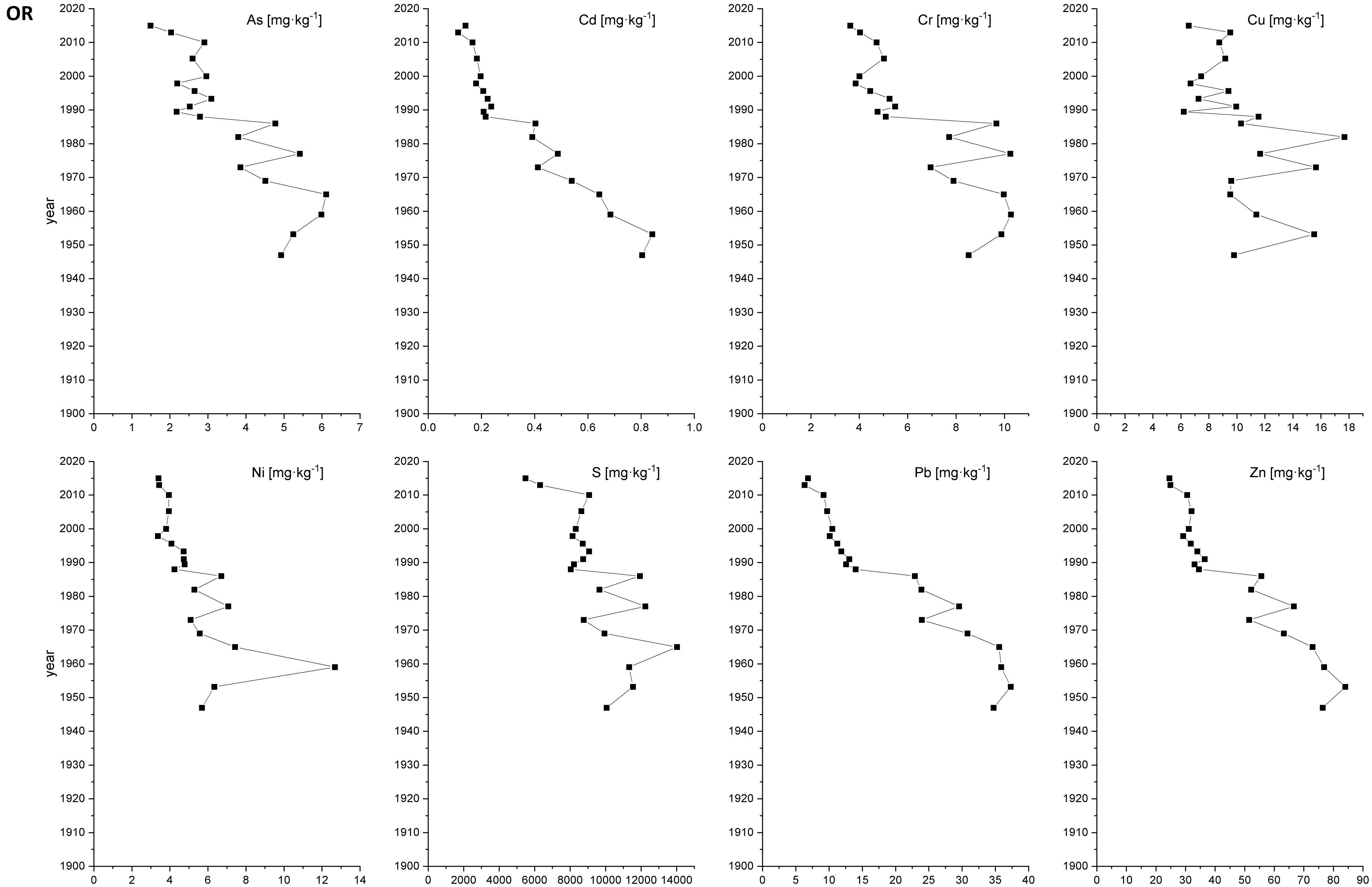

Fig. S10 TE concentrations of the core from Lake Oberucker (OR) in mg·kg^{-1}. Please note the differing x-axes

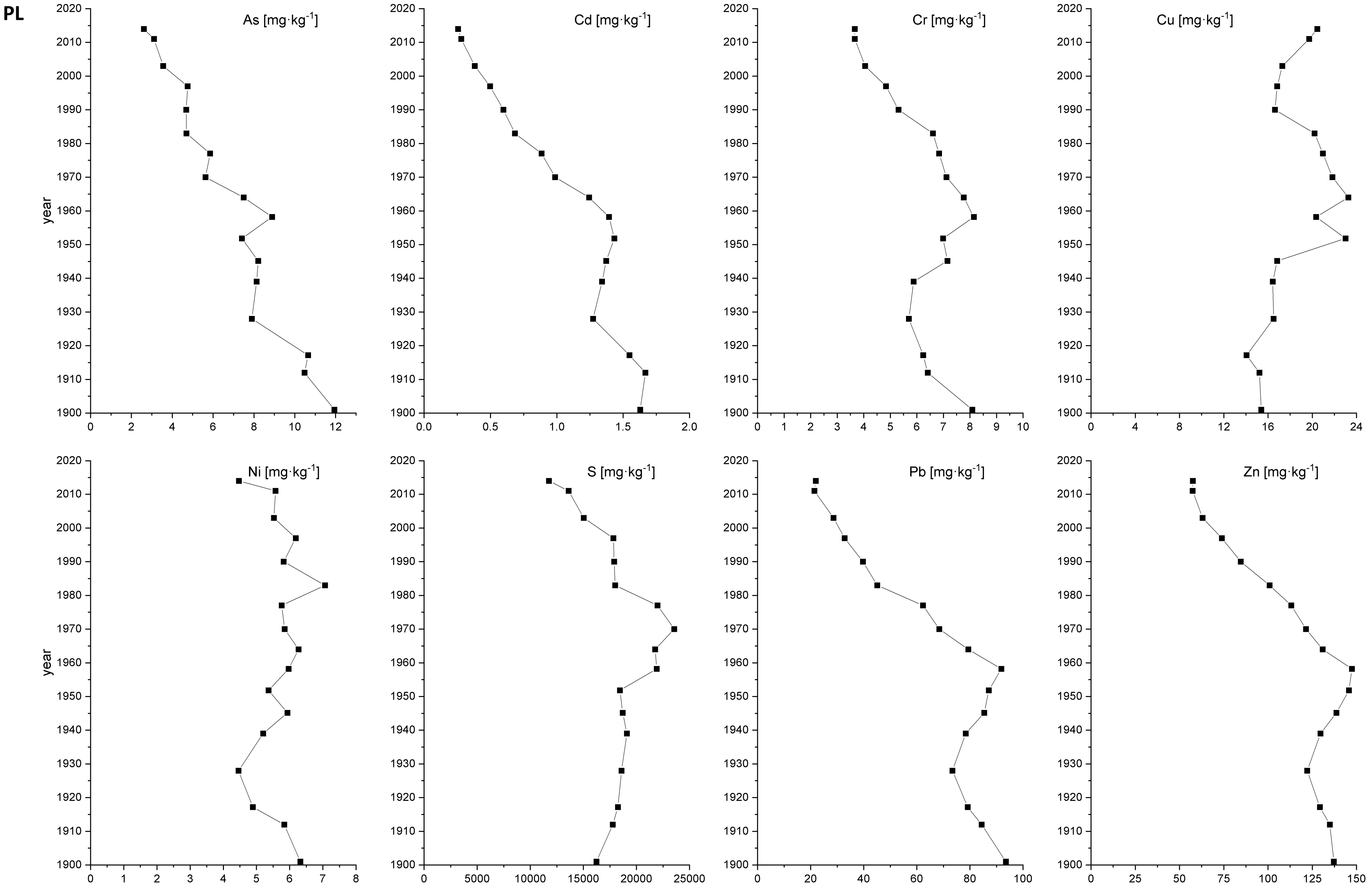

Fig. S11 TE concentrations of the core from Lake Scharmützel (PL) in mg·kg^{-1}. Please note the differing x-axes

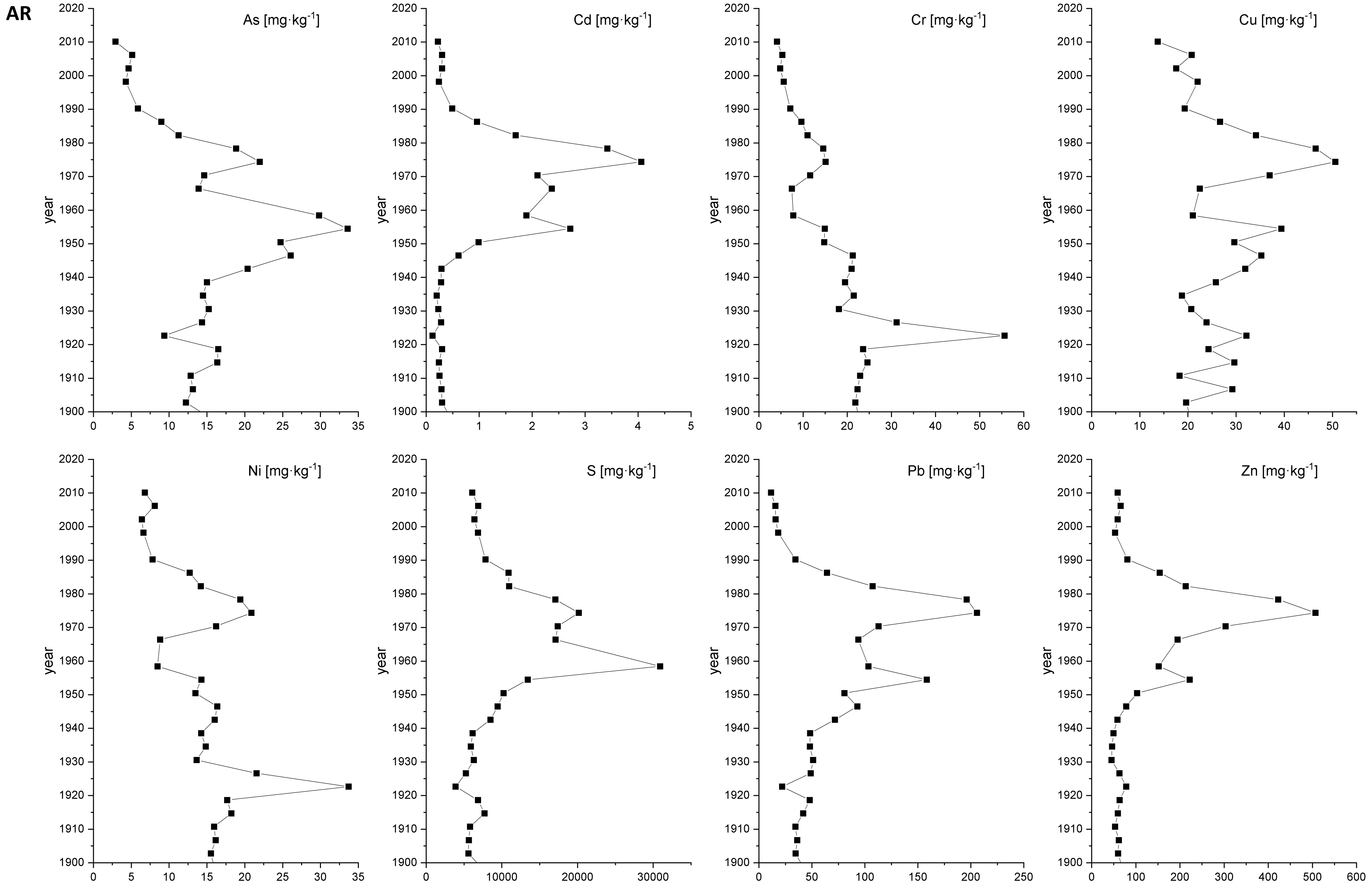

Fig. S12 TE concentrations of the core from Lake Arend (AR) in mg·kg^{-1}. Please note the differing x-axes

ST

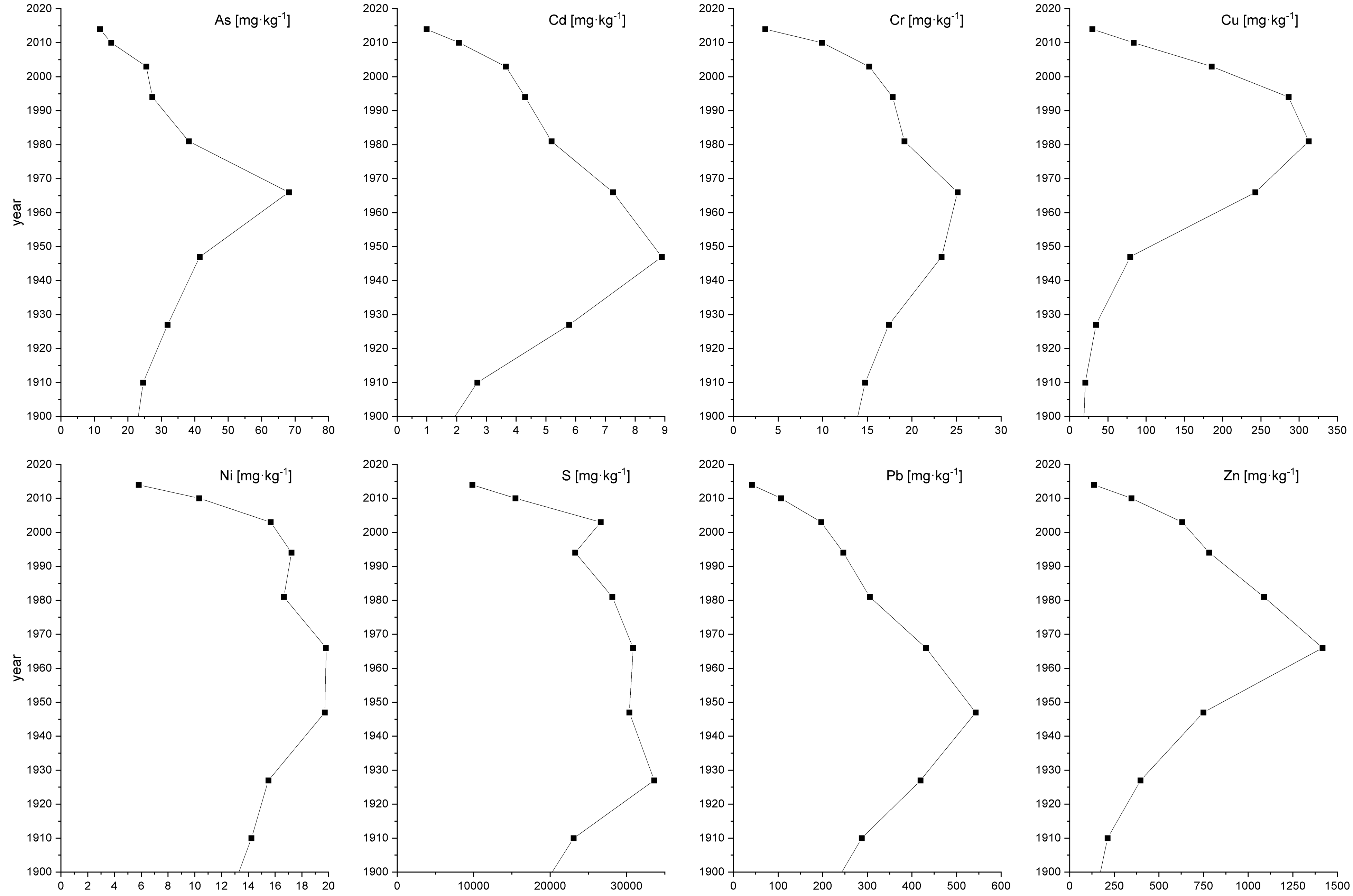

Fig. S13 TE concentrations of the core from Lake Stechlin (ST) in mg·kg^{-1}. Please note the differing x-axes

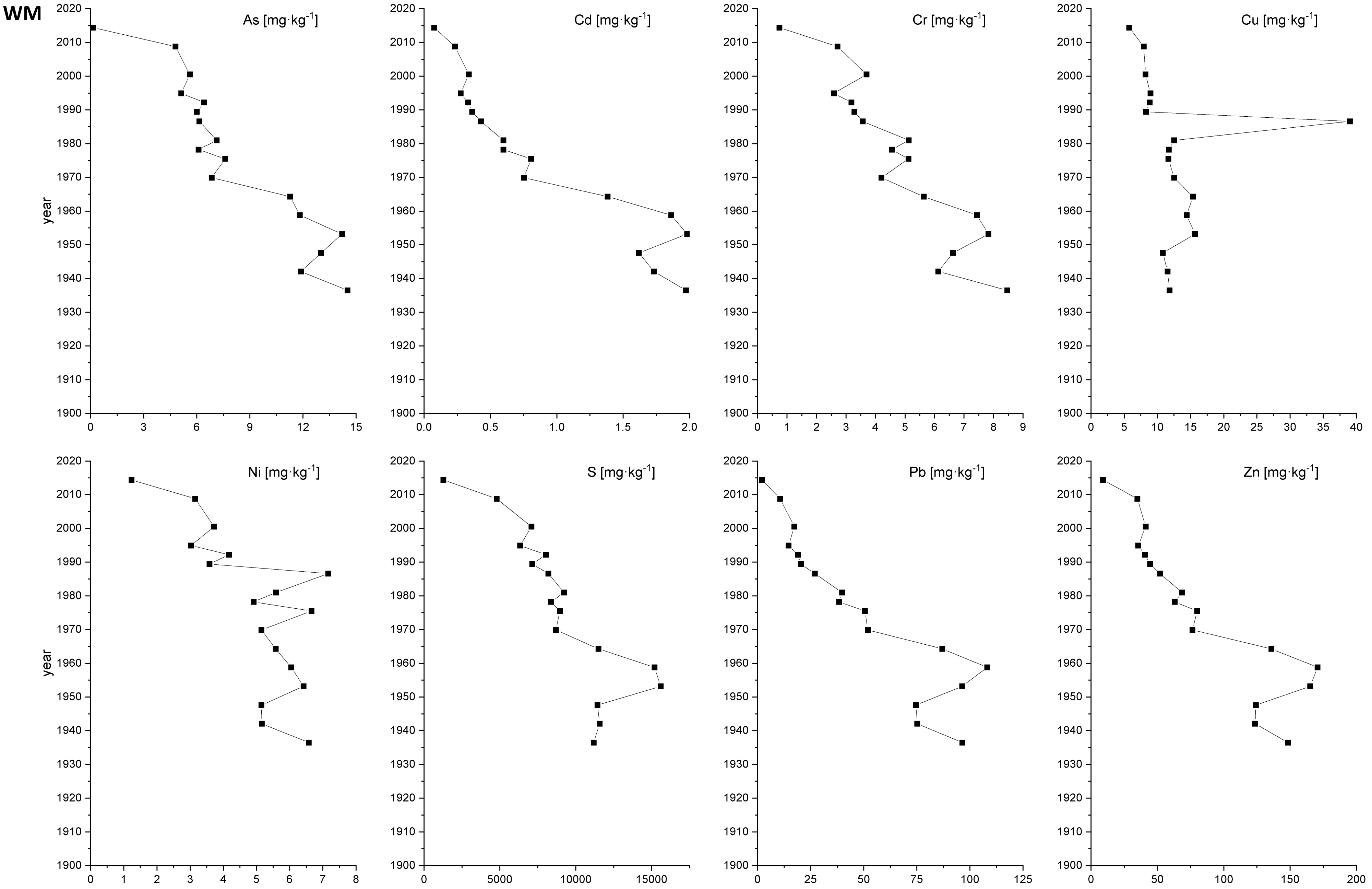

Fig. S14 TE concentrations of the core from Lake Wumm (WM) in mg·kg^{-1}. Please note the differing x-axes

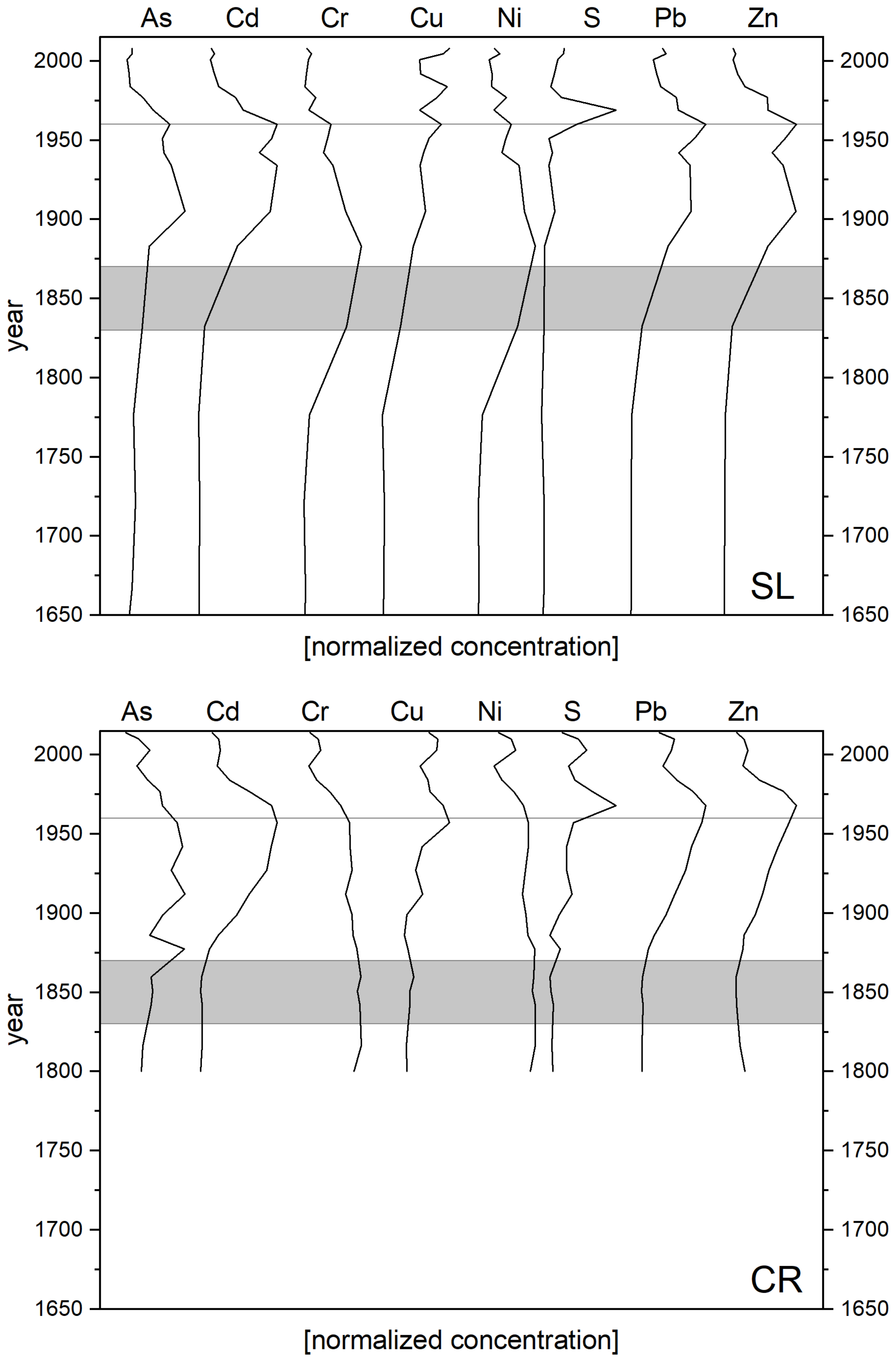

Fig. S15 Normalized TE concentrations of the cores from Lakes Schmaler Luzin (SL) and Carwitzer (CR) that cover time periods before 1900. Gray area shows beginning phase of Industrial Revolution in Germany. Gray line shows the year 1964 when West Germany implemented its first air pollution control regulation

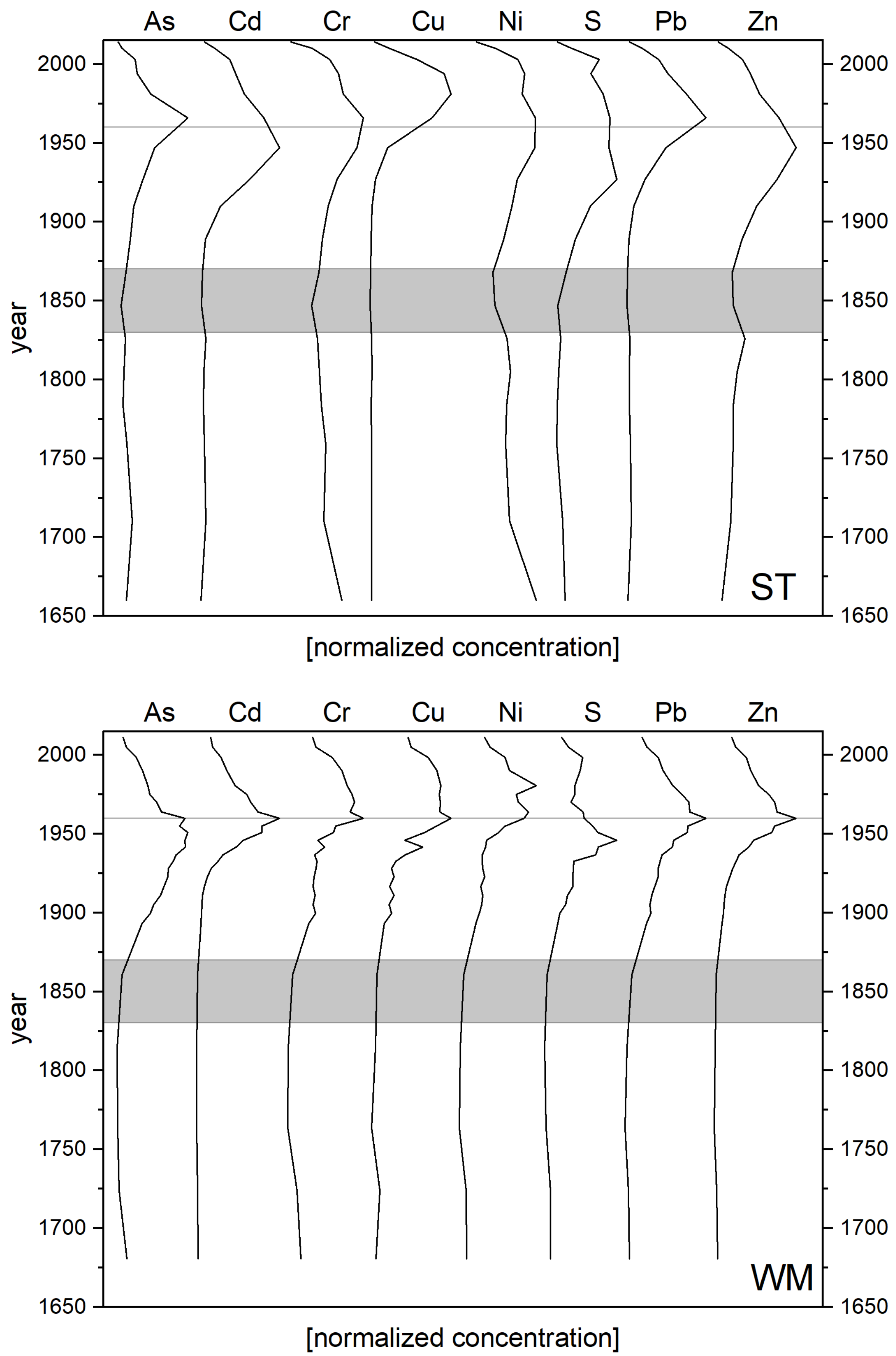

Fig. S16 Normalized TE concentrations of the cores from Lakes Stechlin (ST) and Wumm (WM) that cover time periods before 1900. Gray area shows beginning phase of Industrial Revolution in Germany. Gray line shows the year 1964 when West Germany implemented its first air pollution control regulation

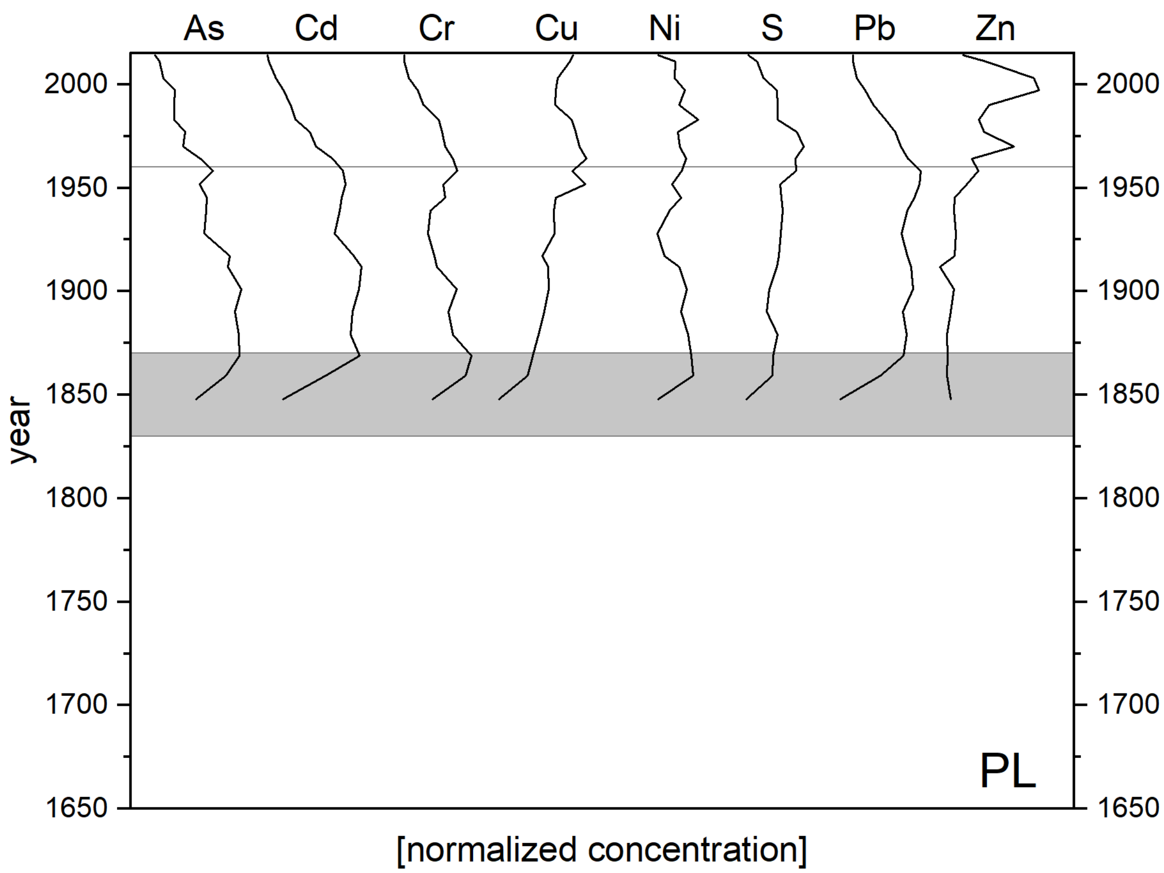

Fig. S17 Normalized TE concentrations of the core from Lake Scharmützel (PL) that covers time periods before 1900. Gray area shows beginning phase of Industrial Revolution in Germany. Gray line shows the year 1964 when West Germany implemented its first air pollution control regulation

References

Rothe M, Kleeberg A, Grüneberg B, Friese K, Pérez-Mayo M, Hupfer M (2015) Sedimentary Sulphur:Iron Ratio Indicates Vivianite Occurrence: A Study from Two Contrasting Freshwater Systems. PLoS ONE 10:e0143737. https://doi.org/10.1371/journal.pone.0143737
